# The efficacy and safety of ginseng berry saponin for heart failure: a systematic review and meta-analysis

**DOI:** 10.3389/fphar.2026.1712401

**Published:** 2026-04-22

**Authors:** Jing Wang, Hongguang Jin, Tianying Chang, Yongsheng Huang, Yingzi Cui

**Affiliations:** 1 School of Traditional Chinese Medicine, Changchun University of Chinese Medicine, Changchun, Jilin, China; 2 Department of Cardiology, the Affiliated Hospital to Changchun University of Chinese Medicine, Changchun, Jilin, China; 3 EBM Office, The Affiliated Hospital to Changchun University of Chinese Medicine, Changchun, Jilin, China

**Keywords:** clinical application, ginseng berry saponin, heart failure, meta-analysis, systematic review

## Abstract

**Background:**

Ginseng berry saponin (GBS), the primary bioactive constituent of *Panax ginseng* C.A. Mey (known as “Renshen” in Chinese) berries, exhibits cardioprotective properties, including anti-inflammatory, antioxidant, and anti-fibrotic effects. In traditional Chinese medicine, they are widely used to treat various cardiovascular diseases. Several randomized controlled trials (RCTs) have shown its efficacy for heart failure (HF).

**Objective:**

To assess the clinical efficacy and safety of GBS as an adjunct therapy for HF through systematic review and meta-analysis.

**Methods:**

A comprehensive and systematic literature search was conducted across seven electronic databases, with no language restrictions, from their respective inception dates through 31 March 2025. These databases included PubMed, the Cochrane Library, EMBASE, Web of Science China National Knowledge Infrastructure China Science and Technology Journal Database (VIP), and Wanfang Data. For quality assessment, the Cochrane Risk of Bias (ROB 2.0) tool was employed, and meta-analyses were performed using Review Manager (RevMan, version 5.4). Under a random-effects model, mean differences and their corresponding 95% confidence intervals (CI) were calculated. Additionally, the certainty of evidence for each outcome was systematically assessed using the GRADE methodology (GRADEpro software v3.6). The study has been registered in PROSPERO, with the registration number CRD420251003193.

**Results:**

The final analysis integrated 32 RCTs, comprising 3,476 HF patients for efficacy and safety assessment. Meta-analysis results indicated that adjunctive GBS therapy significantly improved the following outcomes compared with the control group (all P < 0.01): LVEF (MD = 8.91, 95%CI [6.78, 11.04]), 6MWTD (MD = 63.11, 95%CI [43.27, 82.95]), FS (MD = 2.63, 95%CI [2.04, 3.22]), SV (MD = 6.68, 95%CI [5.56, 7.80]), Cardiac Index (MD = 0.51, 95%CI [0.33, 0.70]), CO (MD = 0.68, 95%CI [0.38, 0.99]), NO (MD = 10.82, 95%CI [7.49, 14.15]), FMD (MD = 2.42, 95%CI [1.45, 3.39]), and NMD (MD = 2.13, 95%CI [1.04, 3.21]). Conversely, adjunctive GBS therapy significantly reduced the following parameters (all P < 0.01): LVEDD (MD = −5.71, 95%CI [−7.59, −3.82]), LVESD (MD = −6.30, 95%CI [−10.00, −2.59]), BNP (MD = −159.86, 95%CI [−199.17, −120.56]), NT-proBNP (MD = −529.13, 95%CI [−673.92, −384.33]), CRP (MD = −1.98, 95%CI [−2.25, −1.71]), hs-CRP (MD = −1.61, 95%CI [−2.66, −0.56]), TNF-α (MD = −20.42, 95%CI [−32.58, −8.26]), MMP-9 (MD = −34.76, 95%CI [−54.96, −14.56]), ET-1 (MD = −20.08, 95%CI [−30.18, −9.98]), SAS score (MD = −7.49, 95%CI [−11.43, −3.55]), SDS score (MD = −14.53, 95%CI [−17.26, −11.80]), HAMA score (MD = −4.48, 95%CI [−6.77, −2.20]), and HAMD score (MD = −5.79, 95%CI [−8.89, −2.68]).

**Conclusion:**

This systematic review suggests that adjunctive GBS therapy may be associated with improvements in surrogate cardiac function measures and patient-reported outcomes in patients with HF. However, these findings should be considered preliminary, as they are derived predominantly from low- and very low-certainty evidence, with no data on hard clinical endpoints such as mortality or hospitalization. Given these substantial limitations, the available evidence does not support the routine clinical use of GBS in HF management. Individualized application may be considered only in the context of shared decision-making and acknowledgment of the underlying evidence uncertainty.

**Systematic Review Registration:**

https://www.crd.york.ac.uk/PROSPERO/view/CRD420251003193.

## 1 Introduction

Heart failure (HF) arises from myocardial impairment, triggering maladaptive cardiac remodeling, characterized by progressive ventricular dysfunction that can advance to end-stage disease in its most severe forms (Lin et al., 2021). While the disease course can vary, it constitutes a mounting global health challenge. This constitutes a mounting global health challenge, affecting over 64 million individuals worldwide with escalating prevalence due to aging populations and improved survival post-acute cardiovascular events (Bozkurt et al., 2021; Conrad et al., 2018; Savarese et al., 2023). Despite optimized guideline-directed medical therapy, which includes renin-angiotensin system inhibitors, beta-blockers, and mineralocorticoid receptor antagonists, a substantial therapeutic gap persists in HF management (Chandra et al., 2019; McMurray et al., 2014). In addition, HF places a heavy burden on people (Heidenreich et al., 2013). HF manifests as a clinical syndrome characterized by cardinal symptoms/signs arising from impaired cardiac structure or function, with diagnostic confirmation requiring elevated natriuretic peptides and/or demonstrable congestion on imaging or hemodynamic monitoring (Bozkurt et al., 2021). Contemporary registries indicate that 40%–50% of patients continue to experience debilitating symptoms (Heidenreich et al., 2022), functional impairment, and reduced quality of life, underscoring the urgent need for novel therapeutic adjuncts (Greene et al., 2018).

Traditional Chinese Medicine (TCM) constitutes a valuable resource for developing novel therapeutics against HF. Among them, the therapeutic effect of Chinese botanical drug medicine *Panax ginseng* C.A. Mey [Araliaceae; Ginseng radix et rhizoma] (known as “Ren shen” in Chinese) is particularly prominent, and it has been widely used in clinical practice for many years (Ma et al., 2017; Xing et al., 2019). *Panax ginseng*, a species of the genus Panax in the Araliaceae family, is widely cultivated in the three northeastern provinces of China (Bai et al., 2025). TCM has a long history of utilizing *P. ginseng* for treating various diseases, particularly those involving qi deficiency and cardiovascular disorders. Its medicinal value has been documented in classical TCM texts for over 2,000 years, forming the basis of its traditional use in managing conditions relevant to modern-day HF. It exhibits therapeutic properties, including tonic effects for physical strengthening, promotion of fluid production to relieve thirst, and tonifying Qi and tranquilizing the mind. Over the years, the rich medicinal value of *P. ginseng* has been well established, and it has both health and medicinal benefits (Pan et al., 2019). The earliest literary records of *P. ginseng* date back to the *Shennong Bencaojing*, a foundational TCM pharmacopoeia compiled around 200 BCE. It classifies *P. ginseng* as a superior botanical drug, noting its effects in replenishing primordial qi, tonifying the spleen and lungs, promoting fluid production, and calming the spirit. These properties align with TCM pathological concepts of HF, which is often attributed to heart qi deficiency (manifesting as fatigue, shortness of breath, and exercise intolerance—symptoms correlating with modern concepts of impaired cardiac output and energy metabolism) or heart yang deficiency (manifesting as cold limbs and edema—symptoms overlapping with low-output syndrome and fluid retention in advanced HF). Later texts, such as the Compendium of Materia Medica (1578 CE) by Shizhen Li, further elaborate on *P. ginseng*’s role in treating heart palpitations due to qi deficiency and chest tightness caused by blood stasis, emphasizing its ability to invigorate qi, circulate blood, stabilize the heart, and calm the mind. These descriptions directly link *P. ginseng* to the management of cardiovascular dysfunction, which is central to HF pathology. In TCM clinical practice, *P. ginseng* has been traditionally used to address core symptoms of HF, either alone or in combination with other botanical drugs. In TCM theory, HF-related edema and congestion are attributed to dampness retention due to impaired qi circulation. *Panax ginseng* is frequently paired with botanical drugs like *Astragalus membranaceus* [Fabaceae; Astragali radix] (known as “Huang qi” in Chinese) and *Poria cocos* [Polyporaceae; Poria] (known as “Fu ling” in Chinese) to promote qi circulation and eliminate dampness, as recorded in the Synopsis of Prescriptions of the Golden Chamber.

The primary medicinal parts of *P. ginseng* include its roots and rhizomes, stems and leaves, berries, and flower buds. The buds, berries, stems, and leaves of ginseng have good pharmacological effects on the cardiovascular, nervous, and immune system, hypoglycemia, anti-tumor, anti-oxidation, anti-aging, and anti-fatigue (Li et al., 2021). While ginseng roots are the most commonly referenced part in classical texts, TCM also recognizes the medicinal value of ginseng berries (known as “Ren shen guo” in Chinese). The *Bencao Congxin* (18th century) notes that ginseng berries have qi-tonifying effects similar to those of the roots but with a stronger ability to clear heat and generate fluids, making them suitable for conditions involving qi deficiency with heat accumulation, which may manifest in HF patients with comorbidities such as anxiety or inflammation. Modern phytochemical studies have identified ginseng berry saponin (GBS) as the primary bioactive constituent of ginseng berries, responsible for their traditional effects. As part of the above-ground portion of the *P. ginseng*, its berry contains approximately four times the saponin content of ginseng roots (F et al., 2013). This linkage connects TCM practice, in which ginseng berries were used to alleviate qi deficiency-related cardiovascular symptoms, with contemporary research on GBS’s cardioprotective mechanisms (e.g., anti-inflammatory, antioxidant, and anti-fibrotic effects), as discussed in this review. It has been found that the major ginsenoside in *P. ginseng* leaves and berries is Rb3 (Wang et al., 2006). It plays a significant role in cardiovascular, pressurizing, anti-myocardial ischemia, anti-arrhythmia, and protection against myocardial injury. In addition, there is a significant therapeutic role in myocardial infarction and depressive co-morbidities (Liu et al., 2019; Liu et al., 2016). Some studies have reported that ginsenosides Rg1 and Rb1 have protective effects in a mouse model of Alzheimer’s disease, and ginsenosides also improve learning and memory in mice (Hou et al., 2020; Hu et al., 2019). Ginseng berry extract has the potential to improve glucose metabolism in humans (Choi et al., 2018; Gao et al., 2024), and its main constituent, ginsenoside Re, has hypoglycemic and anti-obesity effects on obese ob/ob mice and their littermates with lean littermates (Attele et al., 2002; Li et al., 2018). Evidence confirms that anxiety and depression affect 50%–70% of HF patients, with demonstrated negative impacts on prognosis and quality of life (McDonagh et al., 2021; Rutledge et al., 2006). The traditional use of *P. ginseng*—particularly its berries—in treating qi deficiency-related cardiovascular symptoms provides a historical rationale for investigating GBS in HF management. TCM’s emphasis on holistic regulation aligns with the modern recognition that HF requires multi-targeted interventions (e.g., improving cardiac function, reducing inflammation, and enhancing quality of life). By linking traditional applications to GBS’s proven bioactivities, this review strengthens the evidence for translating TCM wisdom into evidence-based HF therapy.

GBS is the main constituent of the patented preparation Zhenyuan Capsule, which has been approved for marketing by the State Food and Drug Administration of China (commercial name as Zhenyuan Capsule, Z22026091). It is effective in benefiting vital energy, opening the veins, tranquilizing the mind, and quenching thirst by generating fluids. Clinically, GBS has been increasingly integrated into HF management protocols across China. A number of randomized controlled trials (RCTs) indicate that adjunctive GBS therapy significantly ameliorates clinical symptoms in HF patients, augments cardiac performance, and reduces the inflammatory response compared with conventional therapy alone. While patient-reported outcomes show significant improvement with GBS, current trials are limited by methodological shortcomings, including small sample sizes, inadequate descriptions of randomization and blinding procedures, high risk of performance and detection bias, and, in many cases, the lack of placebo controls. Absent comprehensive meta-analyses, GBS’s benefit-risk balance remains indeterminate. This study, therefore, undertakes a systematic evaluation of RCTs employing Cochrane methods to establish evidence-based conclusions. This systematic review aims to provide reliable evidence-based guidance for clinical decision-making in HF management.

## 2 Methods

This systematic review and meta-analysis were conducted and reported in accordance with the Preferred Reporting Items for Systematic Reviews and Meta-Analyses (PRISMA) guidelines (Page et al., 2021). As all data were obtained from publicly available databases, no additional ethical approval or patient consent was required. The study protocol was prospectively registered with the International Prospective Register of Systematic Reviews (PROSPERO), registration number CRD420251003193.

### 2.1 Search strategies

We conducted systematic searches in PubMed, Cochrane Library, EMBASE, Web of Science (WOS), China National Knowledge Infrastructure (CNKI), China Science and Technology Journal Database (VIP), and Wanfang Data from their inception through 31 March 2025, without language restrictions. The search strategy utilized relevant Medical Subject Headings (MeSH) and key words, including terms for heart failure (e.g., “Heart Failure,” “Cardiac Failure,” “Heart Decompensation,” “Heart Failure, Left-Sided”) and the intervention (e.g., “ginseng berry saponin,” “American ginseng berry,” “Total Ginseng Fruit Saponins,” “Zhenyuan”). The detailed search strategy is provided in the Supplementary Material.

### 2.2 Study selection

#### 2.2.1 Study design type

This systematic review and meta-analysis included only RCTs that met protocol-defined PICOS criteria.

#### 2.2.2 Patients

Patients with a confirmed diagnosis of HF were enrolled.

#### 2.2.3 Interventions

Eligible HF patients received treatment with GBS preparations, either as monotherapy or as an add-on to conventional HF therapy. The GBS intervention evaluated in all included trials was the same standardized proprietary product: Zhenyuan Capsule (China FDA approval number Z22026091). This preparation is an oral capsule, with a typical dosage of 0.5 g administered three times daily. It contains a standardized extract of total saponins derived from the berry of Panax ginseng (Araliaceae; Ginseng fructus) and is formulated as a single metabolite botanical drug preparation. This consistency confirms the chemical and pharmaceutical comparability of the intervention across all studies.

#### 2.2.4 Control group

The control group received conventional pharmacological therapy, including cardiotonic agents, diuretics, vasodilators, renin-angiotensin system inhibitors (RASIs), β-blockers, and mineralocorticoid receptor antagonists (MRAs), with or without placebo.

#### 2.2.5 Outcomes

Outcomes were stratified by clinical importance using the Grading of Recommendations Assessment, Development and Evaluation (GRADE) framework (Guyatt et al., 2011), with reference to the Clinical Practice Guidelines for the Management of Heart Failure with Chinese Patent Medicine (2021) (Diseases, 2022) and consultation with clinical specialists. The outcomes assessed were categorized as follows: (1). Critical outcomes included clinical events (cardiovascular death or rehospitalization for HF at 6, 12, and 24 months), cardiac structure/function outcomes were left ventricular ejection fraction (LVEF), left ventricular end-diastolic diameter (LVEDD), left ventricular end-systolic diameter (LVESD), and other critical outcomes: B-type natriuretic peptide (BNP), N-terminal pro-BNP (NT-proBNP), 6-min walk test distance (6MWTD). (2). Important (non-critical) outcomes included C-reactive protein (CRP), high-sensitivity CRP (hs-CRP), tumor necrosis factor-α (TNF-α), left ventricular posterior wall thickness (LVPWT), fractional shortening (FS), stroke volume (SV), cardiac index (CI), cardiac output (CO), and adverse events. (3). Not important outcomes were matrix metalloproteinase-9 (MMP-9), nitric oxide (NO), endothelin-1 (ET-1), flow-mediated dilation (FMD), nitroglycerin-mediated dilation (NMD), Self-Rating Anxiety Scale (SAS), Self-Rating Depression Scale (SDS), Hamilton Anxiety Rating Scale (HAMA), and Hamilton Depression Rating Scale (HAMD). This category includes mechanistic biomarkers and patient-reported outcomes of pathophysiological interest that are not yet established as core endpoints for regulatory or clinical decision-making in HF.

### 2.3 Exclusion criteria

The study followed the exclusion criteria: (1) Publications available only as abstracts with inaccessible full texts; (2) Non-journal publications; (3) Excluded due to missing outcome data precluding statistical pooling; (4) Studies of comorbidities with other serious and life-limiting diseases that could independently dominate the prognosis and confound the assessment of HF-specific outcomes, such as active malignancy, severe hepatic or renal failure (e.g., requiring dialysis), or terminal illnesses; (5) Duplicate publications were retained only as the most complete dataset.

### 2.4 Data extraction

Literature records retrieved from the seven electronic databases were managed using EndNote 20 (Clarivate Analytics). Two investigators independently extracted data from eligible studies in adherence to the predefined eligibility criteria, with subsequent cross-checking of all extracted data. Disagreements regarding data extraction were resolved through consultation with a third investigator until consensus was achieved. The following variables were extracted: trial characteristics (author, publication year, sample size); intervention details (treatment duration); patient characteristics (criteria for diagnosis of the disease, age, comorbidities, and, New York Heart Association (NYHA) functional class, and disease duration); primary efficacy outcomes (cardiovascular clinical events, LVEF, LVEDD, LVESD, BNP, NT-proBNP, 6MWTD), secondary outcomes (LVPWT, CRP, hs-CRP, TNF-α, FS, SV, CI, CO, MMP-9, NO, ET-1, FMD, NMD, SAS, SDS, HAMA, HAMD), the information of risk of bias and adverse events.

### 2.5 Assessment of risk of bias

The risk of bias in included studies was assessed using the Cochrane-recommended RoB 2.0 tool. Two independent reviewers assessed risk of bias using the RoB 2.0 tool across five domains: randomization process, deviations from intended interventions, missing outcome data, measurement of the outcome, and selection of the reported result. Studies were categorized as low risk, some concerns, or high risk of bias, with discrepancies resolved through consensus or third-reviewer arbitration.

### 2.6 Statistical analysis and synthesis

Data analysis and synthesis were performed using RevMan5.4 (Cochrane Collaboration). Outcomes data from ≥2 studies were pooled for meta-analysis. Continuous outcomes were analyzed as mean differences (MD) with 95% confidence intervals (CI), while dichotomous data were assessed using risk ratios (RR) with 95% CI. A random-effects model was applied for all meta-analyses, with heterogeneity quantified by I^2^ statistics. Random-effects models were employed for all meta-analyses. When significant heterogeneity was detected (I^2^ ≥ 50% and P ≤ 0.10), we explored potential sources of heterogeneity through sensitivity analyses, subgroup analyses, and meta-regression, where applicable. Subgroup analyses were performed to investigate potential sources of heterogeneity using prespecified variables: baseline comorbidities (e.g., arrhythmias, anxiety/depression) and treatment duration (T < 8 weeks, T ≥ 8 weeks). Formal tests for subgroup differences were conducted to determine whether the effect of GBS differed significantly across subgroups. A P-value<0.05 for the subgroup interaction test was considered indicative of a significant difference in treatment effects between subgroups. Sensitivity analysis employed the leave-one-out method, systematically excluding individual studies sequentially to assess their influence on pooled effect estimates and heterogeneity metrics. Funnel plots were generated to visually assess potential publication bias for outcomes including≥10 studies. For these outcomes, Egger’s linear regression test was also performed in STATA 18.0 to assess funnel plot asymmetry. A p-value<0.05 was considered indicative of significant publication bias.

The certainty of evidence was assessed using the GRADE framework. Evidence quality was evaluated across five downgrade domains: risk of bias (downgraded by one level for moderate risk; two levels for high risk), inconsistency, indirectness, imprecision, and publication bias. Based on these assessments, evidence was categorized into four certainty levels: high, moderate, low, or very low (Balshem et al., 2011). This assessment was independently carried out by two investigators using the GRADE profiler (version 3.6). In case of any disagreements during the process, they could be resolved through discussion or consultation with a third investigator.

## 3 Results

### 3.1 Literature search results

**FIGURE 1:**
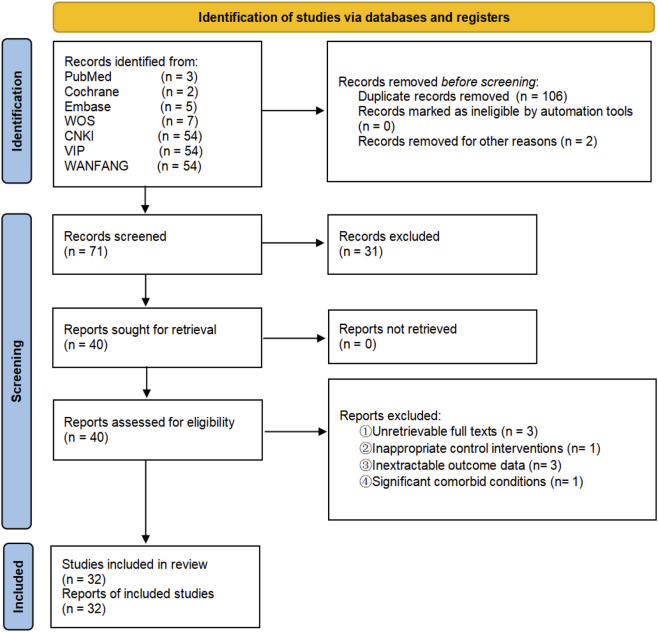
Study screening flow flowchart.

A preliminary search of seven electronic databases yielded 179 records. After deduplication (n = 106) and exclusion of ineligible records (n = 2), 71 records remained. Title/abstract screening excluded 31 studies for: non-RCT designs (n = 23), conference abstracts (n = 6), dissertations (n = 1), and trial protocol (n = 1). Full-text assessment excluded 8 of 40 RCTs for the following reasons: unretrievable full texts (n = 3), inappropriate control interventions (n = 1), inextractable outcome data (n = 3), and significant comorbid conditions (n = 1). Thirty-two RCTs (Cai et al., 2018; Cao et al., 2017; Chen, 2014; Chen et al., 2021; Chen et al., 2015; Dai, 2013; Duan et al., 2014; Fan et al., 2023; He, 2015; Hu and Wang, 2017; Huang and Li, 2019; Lei et al., 2023; Li, 2009; Li et al., 2014; Li et al., 2016; Liao, 2009; Liu et al., 2011; Ma and Hu, 2015; Mi, 2020; Miao, 2019; Wang, 2020; Wang et al., 2018; Wang et al., 2017; Wen et al., 2015; Wu and Jia, 2024; Xu et al., 2017; Ye et al., 2012; Yue et al., 2019; Zhang et al., 2018; Zhao et al., 2019; Zhuang et al., 2018; Zou and Li, 2020) were ultimately included in the meta-analysis. The study selection process is detailed in Figure 1.

### 3.2 Characteristics of included studies

This systematic review and meta-analysis included 32 RCTs, enrolling 3,476 patients with HF (1,784 in the GBS intervention group and 1,692 in the control group). Sample sizes ranged from 52 to 284, with patient ages ranging from 32 to 80 years. Treatment duration ranged from 14 days to 6 months. These trials originated from Chinese research centers, with manuscripts exclusively published in Chinese journals between 2009 and 2024. All trials compared GBS preparations (Zhenyuan Capsule) in combination with conventional drugs for HF with conventional therapy alone. Among the included RCTs: 20 studies (Cai et al., 2018; Chen, 2014; Chen et al., 2015; Duan et al., 2014; Fan et al., 2023; Hu and Wang, 2017; Huang and Li, 2019; Lei et al., 2023; Li, 2009; Li et al., 2014; Liao, 2009; Ma and Hu, 2015; Wang, 2020; Wu and Jia, 2024; Xu et al., 2017; Yue et al., 2019; Zhang et al., 2018; Zhao et al., 2019; Zhuang et al., 2018; Zou and Li, 2020) reported the classification of NYHA heart function (NYHA II: 563 patients; NYHA III: 1,154 patients; NYHA IV: 554 patients), and 18 studies (Cao et al., 2017; Chen, 2014; Hu and Wang, 2017; Huang and Li, 2019; Lei et al., 2023; Li, 2009; Li et al., 2016; Liao, 2009; Ma and Hu, 2015; Miao, 2019; Wang, 2020; Wang et al., 2018; Wu and Jia, 2024; Ye et al., 2012; Yue et al., 2019; Zhao et al., 2019; Zhuang et al., 2018; Zou and Li, 2020) documented HF duration, ranging from 3 months to 25 years. Six studies (Cai et al., 2018; Lei et al., 2023; Mi, 2020; Xu et al., 2017; Zhuang et al., 2018; Zou and Li, 2020) included patients with comorbid arrhythmias (three with bradyarrhythmias; two with ventricular arrhythmias). Five studies (Chen et al., 2015; Li et al., 2016; Wang et al., 2017; Ye et al., 2012; Zhao et al., 2019) enrolled participants with comorbid anxiety and/or depression. Additionally, adverse events were reported in 13 (Cao et al., 2017; Chen et al., 2021; Chen et al., 2015; Dai, 2013; Hu and Wang, 2017; Lei et al., 2023; Wang, 2020; Wu and Jia, 2024; Xu et al., 2017; Ye et al., 2012; Zhang et al., 2018; Zhuang et al., 2018; Zou and Li, 2020) trials, including dizziness, nausea and vomiting, gastrointestinal reactions, fatigue, skin rash, elevated transaminases, and hypotension, et al. No significant serious adverse events occurred in 12 (Cai et al., 2018; Chen, 2014; Duan et al., 2014; Fan et al., 2023; He, 2015; Huang and Li, 2019; Li, 2009; Liao, 2009; Ma and Hu, 2015; Wang et al., 2017; Wen et al., 2015; Yue et al., 2019) studies, while 7 (Li et al., 2014; Li et al., 2016; Liu et al., 2011; Mi, 2020; Miao, 2019; Wang et al., 2018; Zhao et al., 2019) trials reported no adverse events. Detailed characteristics are presented in Table 1.

**TABLE 1:** Characteristics of included studies.

Study	Sample size (Male/Female)		Age (year)		Classification of NYHA heart function (II/III/IV)		Comorbidities (arrhythmia/anxiety/depression)	Course of disease		Intervention(s)		Treatment duration	Outcomes	Adverse events
	T	C	T	C	T	C		T	C	T	C			
Wu and Jia (2024)	52 (29/23)	52 (28/24)	56.11 ± 12.34	55.58 ± 11.46	10/29/13	11/28/13	NA	2.11 ± 0.89 years	2.04 ± 0.85 years	CGI + ZYC 0.5 g tid	RT + sacubitril valsartan sodium tablets 100 mg bid	8 weeks	LVEF, NT-proBNP, BNP, LVEDV, LVESV, 6MWTD, MLHFQ	T: one patient of heart rate slowing; one patient of hypotension C: one patient of arrhythmia; two patients of hypotension; one patient of electrolyte disturbance
Fan, H.J et al. (2023)	53 (31/22)	53 (32/21)	61.67 ± 9.85	62.39 ± 9.95	16/27/10	17/26/10	NA	NA	NA	CGI + ZYC 0.5 g tid	RT + sacubitril valsartan sodium tablets 50–100 mg bid (maximum dose increased to 200 mg bid)	16 weeks	LVEF, NT-proBNP, LVEDD, LVPWT, IVST	No occurred
Lei, R et al. (2023)	58 (32/26)	58 (28/30)	62.91 ± 9.97	61.32 ± 9.56	0/27/31	0/23/35	Combined arrhythmia	12.37 ± 2.97 months	11.65 ± 2.90 months	CGI + GBS preparation (ZYC) 0.5 g tid	Hydrochlorothiazide tablet 25 mg bid, enalapril maleate tablets 10 mg qd, digoxin 0.2 mg qd, metoprolol succinate sustained-release tablets 47.5 mg qd, calcium dibutyryladenosine cyclophosphate for injection 40 mg qd	12 weeks	LVEF, SV, CO, CI, DPV, SPV, CBF, CR, ET-1, MMP-9	T: three patients of nausea and vomiting; two patients of diarrhea and abdominal pain; two patients of drowsiness; two patients of skin rash C: two patients of nausea and vomiting; two patients of diarrhea and abdominal pain; one patient of drowsiness; one patient of skin rash
Chen, X et al. (2021)	30 (15/15)	30 (16/14)	55.26 ± 4.52	56.3 ± 3.58	NA	NA	NA	NA	NA	CGI + ZYC 0.5 g tid	RT + trimetazidine 20 mg tid	2 weeks	LVEF, BNP, LVESD, LVEDD, TNF-α, hs-CRP	T: one patient of allergy; one patient of nausea C: two patients of allergy; one patient of chest tightness; one patient of nausea
Zou and Li (2020)	51 (29/22)	51 (28/23)	58.58 ± 6.26	58.54 ± 7.19	0/32/19	0/34/17	Combined bradyarrhythmias	4.47 ± 0.53 years	4.32 ± 0.69 years	CGI + GBS preparation (ZYC) 0.5 g tid	RT + calcium dibutyryladenosine cyclophosphate for injection 40 mg qd	T: 4 weeks C: 3 weeks	LVEF, ET-1, MMP-9	T: two patients of abdominal distention; four patients of dizziness; three patients of nausea C: three patients of abdominal distention; four patients of dizziness; three patients of nausea
Wang, C.L (2020)	97 (46/51)	96 (47/49)	65.72 ± 5.95	65.48 ± 4.28	31/36/30	29/35/32	NA	5.29 ± 1.28 years	5.37 ± 1.12 years	CGI + ZYC 0.5 g tid	RT + trimetazidine 20 mg tid	1 month	LVEF, BNP, LVESD, LVEDD, CRP, MLHFQ	T: five patients of palpitations; five patients of dizziness; three patients of elevated transaminases C: four patients of palpitations; four patients of dizziness; three patients of elevated transaminases
Mi, S.W (2020)	38 (19/19)	38 (21/17)	63.8 ± 6.2	64.3 ± 5.8	NA	NA	Combined ventricular arrhythmia	NA	NA	RT + GBS 0.5 g tid	RT + amiodarone	12 weeks	LVEF, FMD, NMD, NO, 6MWTD	Not reported
Yue, Z.Z et al. (2019)	60 (34/26)	60 (32/28)	63.18 ± 4.75	62.65 ± 4.87	21/32/7	22/30/8	NA	6.49 ± 2.28 years	6.52 ± 2.36 years	CGI + ZYC 0.5 g tid	RT	8 weeks	LVEF, NT-proBNP, HAMD, HAMA	No occurred
Huang and Li (2019)	40 (14/26)	40 (15/25)	32–72	33–71	12/22/6	10/23/7	NA	5–25 years	5–24 years	RT + ZYC 0.5 g tid	RT	30 days	LVEF	No occurred
Miao L (2019)	63 (39/24)	63 (37/26)	59.41 ± 7.58	58.62 ± 8.01	NA	NA	NA	6.03 ± 1.84 years	5.98 ± 2.01 years	CGI + ZYC 0.5 g tid	Cardiac stimulants, diuretics, aspirin, vasoconstrictor tensin-converting enzyme inhibitors + metoprolol 7 mg bid (maximum dose not to exceed 80 mg)	4 weeks	LVEF, LVESD, LVEDD, TNF-α, hs-CRP	Not reported
Zhao, W.P et al. (2019)	40 (19/21)	40 (21/19)	65.32 ± 13.21	65.28 ± 13.37	14/22/4	13/21/6	With anxiety and depression	7.17 ± 2.58 years	7.37 ± 2.18 years	RT + ZYC 0.5 g tid	RT	30 days	SAS, SDS	Not reported
Cai, H.Z et al. (2018)	41 (24/17)	41 (22/19)	64.4 ± 7.1	63.9 ± 7.6	0/28/13	0/27/14	Combined ventricular arrhythmia	NA	NA	CGI + GBS 0.5 g tid	RT + amiodarone	12 weeks	LVEF, LVESD, LVEDD, CO, TXB2, ET-1, FMD, NMD, NO	No occurred
Zhuang, H et al. (2018)	46 (27/19)	46 (26/20)	58.6 ± 6.5	58.4 ± 7.1	0/30/16	0/32/14	Combined bradyarrhythmias	4.7 ± 0.6 years	4.4 ± 0.7 years	CGI + GBS (ZYC) 0.5 g tid	RT + calcium dibutyryladenosine cyclophosphate for injection 40 mg	2 weeks	LVEF, ET-1, MMP-9, HR	T: three patients of nausea; three patients of dizziness; one patient of abdominal distention C: two patients of nausea; four patients of dizziness; two patients of abdominal distention
Zhang, R.S et al. (2018)	108 (71/37)	108 (72/36)	55.3 ± 12.3	56.2 ± 13.2	25/72/11	26/70/12	NA	NA	NA	CGI + ZYC 0.5 g tid + XYC 1.2 g tid	RT	3 months	LVEF, 6MWTD	T: two patients of fatigue C: thirteen patients of fatigue
Wang, D et al. (2018)	54 (32/22)	54 (34/20)	56.9 ± 8.6	57.1 ± 7.2	NA	NA	NA	5.6 ± 3.1 years	5.5 ± 2.9 years	CGI + ZYC 0.5 g tid	RT + adenosine cyclophosphate for injection 40 mg qd	2 weeks	LVEF, BNP, FS, TNF-α, hs-CRP	Not reported
Cao, F et al. (2017)	142 (76/66)	142 (69/73)	36.34 ± 17.28	38.02 ± 17.22	NA	NA	NA	3.18 ± 2.26 years	3.29 ± 1.98 years	CGI + ZYC 0.5 g tid	RT + adenosine cyclophosphate for injection 35 mg qd	30 days	LVEF, BNP, LVESD, LVEDD, CRP, 6MWTD	T: eight patients of dizziness; six patients of headache; five patients of abdominal distention; seven patients of fatigue; five patients of nausea C: twenty-one patients of dizziness; nineteen patients of headache; twenty-six patients of abdominal distention; twenty-three patients of fatigue; eighteen patients of nausea
Xu, H.M et al. (2017)	Group 1 80 (47/33) Group 2 80 (40/40)	80 (43/37)	Group 1 67 ± 11 Group 2 68 ± 11	68 ± 12	Group 1 0/30/50 Group 2 0/36/44	0/33/47	Combined bradyarrhythmias	NA	NA	CGI + GBS preparation 0.5 g tid + calcium dibutyryladenosine cyclophosphate for injection 40 mg qd	RT	T: Group 1 4 weeks Group 2 8 weeks C: 4 weeks/8 weeks	LVEF, BNP, ET-1, MMP-9, 6MWTD	T: four patients of dizziness, nausea, abdominal distention C: not reported
Hu and Wang (2017)	63 (34/29)	63 (35/28)	65.39 ± 12.46	64.93 ± 11.85	31/19/13	32/19/12	NA	4.28 ± 1.35 years	4.32 ± 1.57 years	CGI + ZYC 1 g tid	RT + meglumine adenosine cyclphosphate injection 120 mg qd	4 weeks	LVEF, NT-proBNP, LVEDD, LVPWT	T: four patients of nausea; four patients of stomach upset; one patient of bradycardia C: four patients of nausea; five patients of stomach upset; one patient of bradycardia
Wang, F.Y et al. (2017)	43 (29/14)	43 (25/18)	67.5 ± 5.6	68.9 ± 6.2	NA	NA	With anxiety	NA	NA	CGI + ZYC 0.5 g tid	RT + lorazepam 0.5 mg bid	6 weeks	LVEF, NT-proBNP, FS, SAS	No occurred
Li, L et al. (2016)	30 (16/14)	30 (18/12)	56.25 ± 7.41	57.42 ± 7.63	NA	NA	With anxiety and depression	8.12 ± 2.21 years	7.98 ± 2.82 years	CGI + ZYC 0.5 g tid	RT	30 days	SAS, SDS	Not reported
Wen, J et al. (2015)	53	52	55 ± 4	54 ± 3	NA	NA	NA	NA	NA	CGI + ZYC 0.5 g tid	RT + trimetazidine 20 mg tid	3 months	LVEF, NT-proBNP, SV, CO, CI, 6MWTD	No occurred
He, P.H (2015)	60	60	NA	NA	NA	NA	NA	NA	NA	RT + ZYC 0.5 g tid	RT	8 weeks	LVEF, NT-proBNP, LVEDD, SV, CO, CI hs-CRP, 6MWTD	No occurred
Chen et al. (2015)	41 (22/19)	40 (18/22)	73.8 ± 5.7	74.4 ± 5.6	9/27/5	12/21/7	With anxiety	NA	NA	CGI + ZYC 0.5 g tid	RT + lorazepam 0.5 mg bid	4 weeks	LVEF, NT-proBNP, SAS, HAMA	Fewer adverse reactions; only three patients of daytime sleepiness were seen in both groups
Ma, S et al., 2015	36 (21/15)	32 (18/14)	67.8 ± 12.1	63.7 ± 13.2	15/19/2	13/17/2	NA	5.7 ± 1.8 years	6.0 ± 1.2 years	RT + ZYC 0.5 g tid	RT	4 weeks	BNP	No occurred
Li, K et al. (2014)	42	42	NA	NA	21/48/15		NA	NA	NA	RT + ZYC 0.75 g tid	RT	6 months	LVEF, NT-proBNP	Not reported
Duan, X.Z et al. (2014)	42 (24/18)	42 (25/17)	65.1 ± 7.8	63.7 ± 8.5	11/24/7	10/25/7	NA	NA	NA	RT + ZYC 0.5 g tid	RT	3 months	LVEF, LVEDD, 6MWTD	No occurred
Chen, C.G (2014)	45 (24/21)	44 (24/20)	60.7 ± 13.5	61.5 ± 12.8	26/19/0	26/18/0	NA	5.4 ± 1.3 years	5.2 ± 1.1 years	CGI + ZYC 0.5 g tid	RT (digoxin tablets 0.125 mg qd, hydrochlorothiazide tablets 25 mg qd 3 days/week)	4 weeks	LVEF, CO	No occurred
Dai, C.J (2013)	32 (19/13)	32 (20/12)	67.1 ± 11.3	66.8 ± 10.9	NA	NA	NA	NA	NA	RT + ZYC 0.5 g tid	RT + acarbose tablets 50 mg tid	6 months	LVEF, NT-proBNP, FPG, 2hPG, HbA1c	T: no occurred C: two patients of abdominal distention
Ye, Q.H et al. (2012)	37 (20/17)	37 (20/17)	66 ± 9	67 ± 8	NA	NA	With anxiety and depression	3.6 ± 2.4 months	3.4 ± 2.9 months	CGI + ZYC 0.5 g bid or tid	RT + paroxetine hydrochloride tablets	6 weeks	LVEF, FS, HAMD, HAMA	T: two patients of hypertension; two patients of dizziness and headache; six patients of nausea and vomiting; two patients of inability to sit still; nine patients of dry mouth; five patients of constipation; three patients of blurred vision; two patients of insomnia; two patients of chest tightness and palpitation; three patients of blood abnormalities; three patients of hepatic abnormalities; two patients of electrocardiogram abnormalities C: four patients of hypertension; four patients of dizziness and headache; eight patients of nausea and vomiting; three patients of inability to sit still; eleven patients of dry mouth; six patients of constipation; five patients of blurred vision; eleven patients of insomnia; thirteen patients of chest tightness and palpitation; five patients of blood abnormalities; ten patients of hepatic abnormalities; thirteen patients of electrocardiogram abnormalities
Liu, Z.G et al. (2011)	28	24	NA	NA	NA	NA	NA	NA	NA	Benazepril hydrochloride Tablets 10 mg qd + ZYC 0.25 g tid	Benazepril hydrochloride Tablets 10 mg qd	6 months	LVEF, LVESD, LVEDD, 6MWTD	Not reported
Li, D.F (2009)	60 (32/28)	60 (27/33)	60.5	66.51	28/25/7	25/27/8	NA	4.8 years	4.52 years	RT + ZYC 1 g tid	RT	14 days	LVEF, SV, FS, CO	No occurred
Liao, Y.X (2009)	39 (19/20)	39 (23/16)	46 ± 2.3	46 ± 2.1	24/15/0	23/26/0	NA	6 ± 0.7 years	6 ± 0.5 years	RT + ZYC 0.5 g tid	RT	28 days	LVEF	No occurred

T, intervention group; C, control group; CGI, control group interventions; GBS, ginseng berry saponin; ZYC, zhenyuan capsule; XYC, xinyuan capsule; RT, routine treatment; qd, once daily; bid, twice daily; tid, three times daily; NA, data missing; LVEF, left ventricular ejection fraction; BNP, B-type natriuretic peptide; NT-proBNP, N-terminal pro-BNP; 6MWTD, 6-min walk test distance; LVEDV, left ventricular end-diastolic volume; LVEDD, left ventricular end-diastolic diameter; LVESD, left ventricular end-systolic diameter; LVESV, left ventricular end-systolic volume; LVPWT, left ventricular posterior wall thickness; IVST, interventricular septal thickness; FS, fractional shortening; SV, stroke volume; CO, cardiac output; CI, cardiac index; DPV, diastolic peak velocity; SPV, systolic peak velocity; CBF, coronary blood flow; CR, coronary resistance; TNF-α, Tumor necrosis factor α; CRP, C-reactive protein; hs-CRP, hypersensitive C-reactive protein; ET-1, Endothelin-1; TXB2, Thromboxane B2; FMD, brachial flow-mediated dilatation; NMD, Nitroglycerin-mediated dilation; NO, nitric oxide; MMP-9, Matrix metallopeptidase-9; HR, heart rate; MLHFQ, Minnesota Living with Heart Failure Questionnaire; SAS, Self-Rating Anxiety Scale; SDS, Self-rating depression scale; HAMA, hamilton anxiety scale; HAMD, hamilton depression scale; FPG, fasting plasma glucose; 2hPG, 2-h plasma glucose; HbA1c, glycated hemoglobin.

### 3.3 Risk of bias assessment results

**FIGURE 2:**
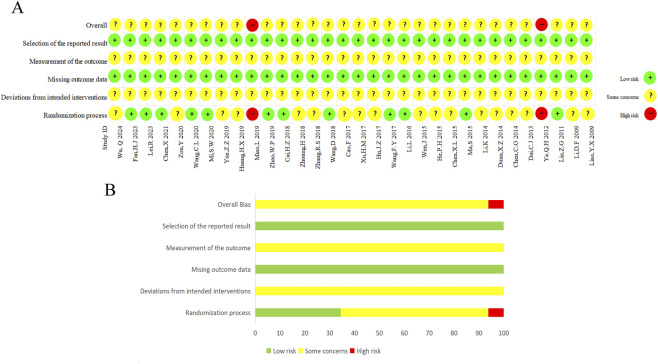
Risk of bias assessment results for trials included in this meta-analysis. **(A)** Risk of bias summary for each included study. **(B)** Risk of bias graph for each domain for all included studies.

Two researchers independently conducted risk of bias assessments with cross-verification. Results are presented in Figure 2. Among the 32 included RCTs, 2 studies (Miao, 2019; Ye et al., 2012) were judged to have a high ROB, while 30 trials (Cai et al., 2018; Cao et al., 2017; Chen, 2014; Chen et al., 2021; Chen et al., 2015; Dai, 2013; Duan et al., 2014; Fan et al., 2023; He, 2015; Hu and Wang, 2017; Huang and Li, 2019; Lei et al., 2023; Li, 2009; Li et al., 2014; Li et al., 2016; Liao, 2009; Liu et al., 2011; Ma and Hu, 2015; Mi, 2020; Wang, 2020; Wang et al., 2018; Wang et al., 2017; Wen et al., 2015; Wu and Jia, 2024; Xu et al., 2017; Yue et al., 2019; Zhang et al., 2018; Zhao et al., 2019; Zhuang et al., 2018; Zou and Li, 2020) presented some concerns in the overall RoB assessment. The high-risk trials had inadequate descriptions of randomization methods and complete absence of blinding or placebo use across all assessed procedures. In two key domains (missing outcome data, and selection of the reported result), a low risk was shown in all thirty-two studies. Since all included studies relied on outcome measures such as LVEF, 6MWTD, and anxiety/depression scales, and did not specify whether they used blinded echocardiography or a central outcome adjudication mechanism for these assessments, the outcome measurement domain (Domain 4) was rated as having some concerns. The concerns identified in deviations from intended interventions (Domain 2), primarily due to unreported blinding procedures, seriously undermine the robustness of the pooled effect estimates and likely lead to an overestimation of the treatment benefits.

### 3.4 Efficacy analysis results

#### 3.4.1 Primary outcomes

##### 3.4.1.1 Cardiovascular clinical events

None of the included RCTs reported cardiovascular death or rehospitalization for HF at 6, 12, and 24-month follow-up timepoints.

##### 3.4.1.2 Efficacy on cardiac structure and function

1. Left ventricular ejection fractions (LVEF)

**FIGURE 3:**
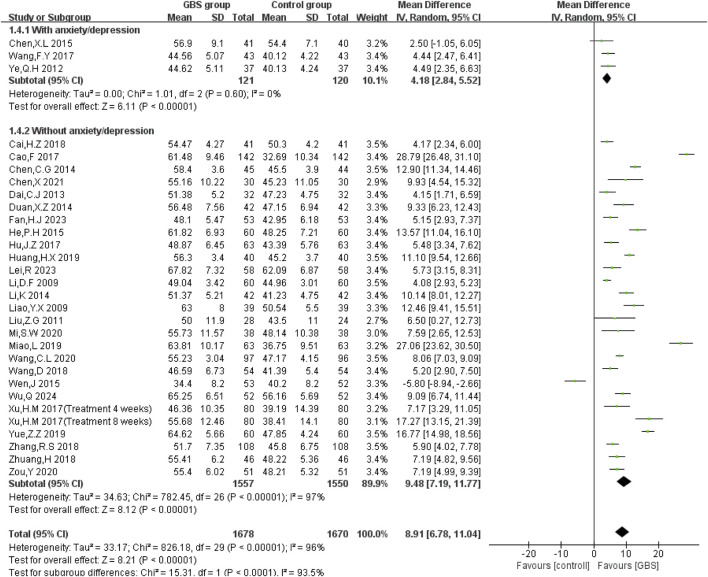
Anxiety/depression subgroup analysis of the effect of GBS on LVEF.

LVEF, a critical outcome for assessing cardiac function, was reported in 29 studies. Random-effects models were used to analyze pooled data (MD = 8.91, 95% CI [6.78, 11.04], P < 0.00001) with significant heterogeneity (P < 0.00001, I^2^ = 96%). Compared with the control group, the meta-analysis showed that the GBS intervention group had a significantly higher LVEF. Heterogeneity still existed after subgroup analysis by treatment duration (T < 8 weeks, T ≥ 8 weeks; P for interaction = 0.35) and in patients with comorbid arrhythmias (P for interaction = 0.43; presented in Supplementary Figure S1). However, reduced heterogeneity (P for interaction<0.0001) in the anxiety/depression subgroup suggests psychological status may contribute to heterogeneity (Figure 3).

2. Left ventricular end-diastolic diameter (LVEDD)

**FIGURE 4:**
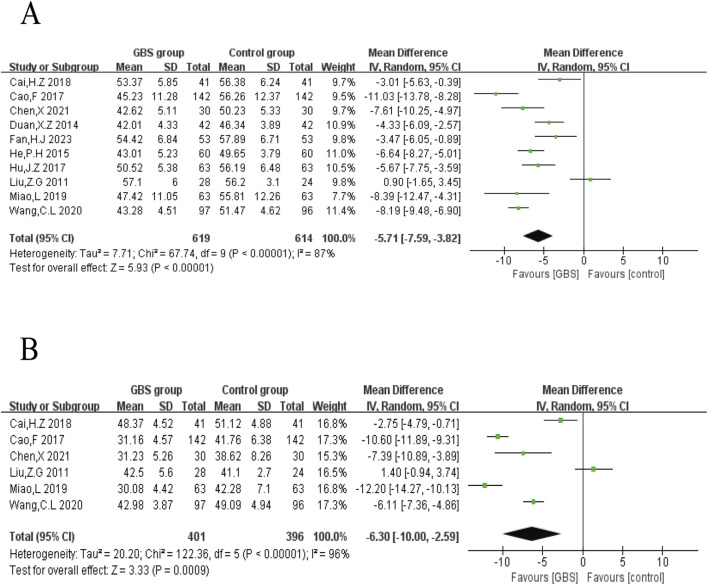
Meta-analysis of the effects of GBS on **(A)** LVEDD, **(B)** LVESD.

In ten studies, LVEDD was evaluated. The results showed significant heterogeneity among these studies (P < 0.00001, I^2^ = 87%). The results of meta-analysis under a random-effects model showed that LVEDD was lower in the GBS intervention group (n = 1,233, MD = −5.71, 95% CI [−7.59, −3.82], P < 0.00001) (Figure 4A).

3. Left ventricular end-systolic diameter (LVESD)

LVESD was reported in six studies. Random-effects models were used to analyze data (n = 797, MD = −6.30, 95% CI [−10.00, −2.59], P = 0.0009) for significant heterogeneity (P < 0.00001, I^2^ = 96%). Meta-analysis showed that GBS preparations were more significant in reducing LVESD (Figure 4B).

##### 3.4.1.3 Efficacy on BNP and NT-proBNP

1. B-type natriuretic peptide (BNP)

**FIGURE 5:**
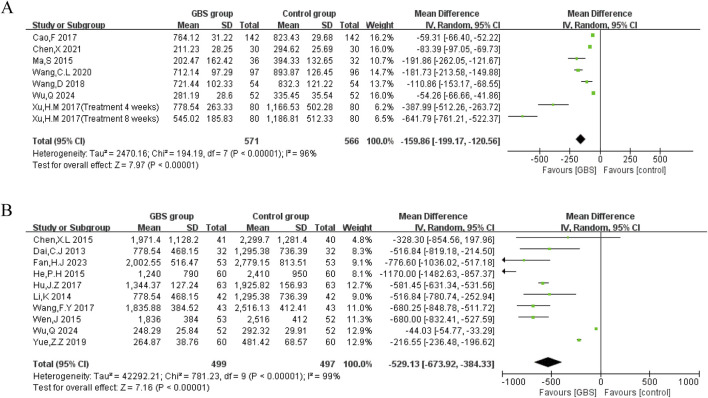
Meta-analysis of the effects of GBS on **(A)** BNP, **(B)** NT-pro-BNP.

BNP (measurement unit: pg/mL) was reported in seven studies. Meta-analysis under a random-effects model showed significant heterogeneity (P < 0.00001, I^2^ = 96%). The GBS preparation intervention group demonstrated a significant reduction in BNP compared with the control group (MD = −159.86, 95% CI [−199.17, −120.56], P < 0.00001) (Figure 5A).

2. N-terminal pro-BNP (NT-proBNP)

Ten studies reported NT-proBNP (measurement unit: pg/mL). Meta-analysis showed a statistically significant reduction in NT-proBNP in the GBS intervention group (n = 996, MD = −529.13, 95% CI [−673.92, −384.33], P < 0.00001). The results showed significant heterogeneity among these studies (P < 0.00001, I^2^ = 99%) (Figure 5B).

##### 3.4.1.4 Efficacy on 6-min walk test distance (6MWTD)

**FIGURE 6:**
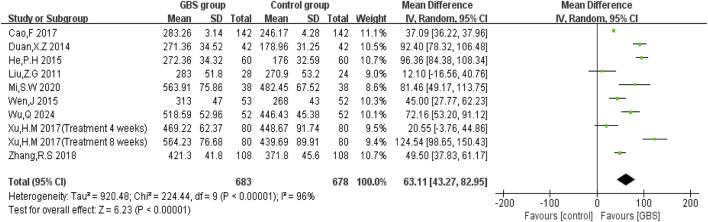
Forest plot of impacts of GBS on 6MWTD.

We were able to pool results from 9 studies that evaluated the effect of the GBS intervention group against 6MWTD. Random-effects models were used to analyze pooled data (MD = 63.11, 95% CI [43.27, 82.95], P < 0.00001) with significant heterogeneity (P < 0.00001, I^2^ = 96%) (Figure 6).

#### 3.4.2 Secondary outcomes

##### 3.4.2.1 Efficacy on the structure and function of the heart

1. Left ventricular posterior wall thickness (LVPWT)

**FIGURE 7:**
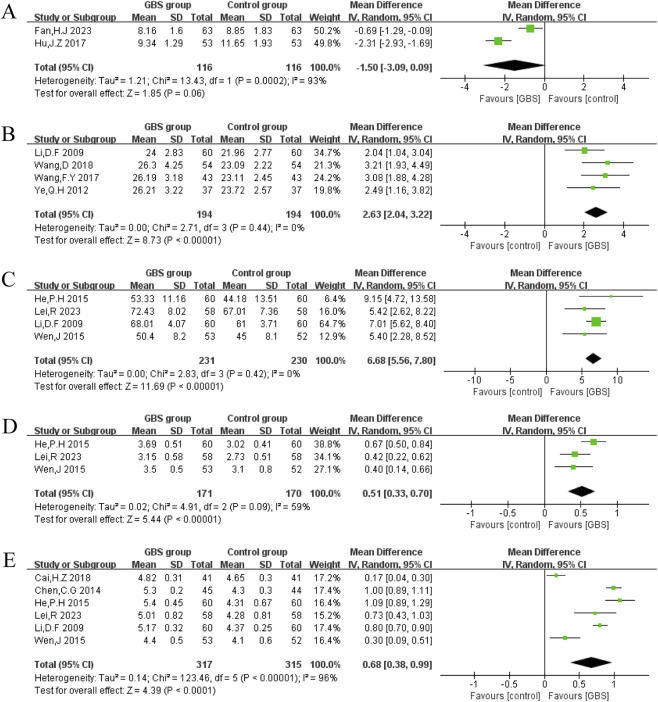
Forest plot of impacts of GBS on **(A)** LVPWT, **(B)** FS, **(C)** SV, **(D)** CI, and **(E)** CO.

Only two studies observed changes in LVPWT. Meta-analysis showed no statistically significant difference in LVPWT reduction in the GBS preparations intervention group (n = 232, MD = −1.50, 95% CI [−3.09, 0.09], P = 0.06), with significant heterogeneity (P = 0.0002, I^2^ = 93%) (Figure 7A).

2. Fractional shortening (FS)

FS was reported by four studies. Meta-analysis showed a statistically significant difference in FS in the GBS preparations intervention group (n = 388, MD = 2.63, 95% CI [2.04, 3.22], P < 0.00001), with no significant heterogeneity (P = 0.44, I^2^ = 0%). Meta-analysis showed that the GBS group increased FS more than the control group (Figure 7B).

3. Stroke volume (SV)

SV was reported in four studies. It was significantly higher in the GBS group than in the control group (n = 461, MD = 6.68, 95% CI [5.56, 7.80], P < 0.00001). Meta-analysis under a random-effects model showed no significant heterogeneity (P = 0.42, I^2^ = 0%) (Figure 7C).

4. Cardiac index (CI)

CI was reported by three studies. Random-effects models were used to analyze pooled data (n = 341, MD = 0.51, 95% confidence intervals (CI) [0.33, 0.70], P < 0.00001) for significant heterogeneous distribution (P = 0.09, I^2^ = 59%). Based on meta-analysis, the GBS intervention increased CI more effectively than the control group (Figure 7D).

5. Cardiac output (CO)

Six studies evaluated CO, and significant heterogeneity was observed (P < 0.00001, I^2^ = 96%). A random-effects model was used, and the meta-analysis showed that CO was higher in the GBS preparations intervention group (n = 632, MD = 0.68, 95% CI [0.38, 0.99], P < 0.00001) (Figure 7E).

##### 3.4.2.2 Efficacy on indicators of inflammation

1. C-reactive protein (CRP)

**FIGURE 8:**
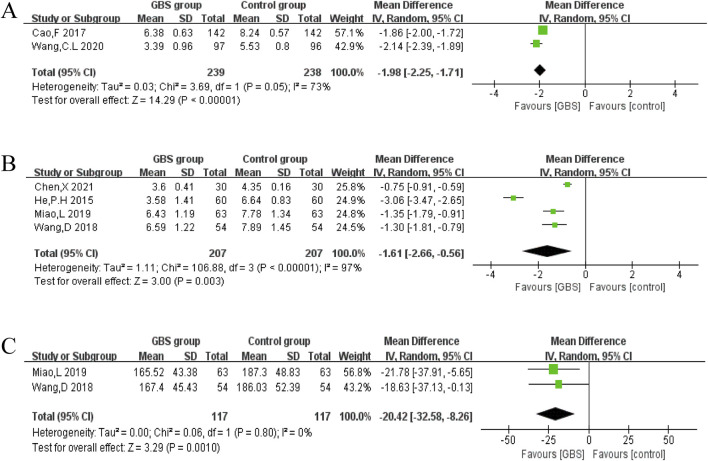
Effects of GBS on **(A)** CRP, **(B)** hs-CRP and **(C)** TNF-α.

In two studies, CRP was reported. A random-effects model was constructed, and the meta-analysis results showed that CRP was lower in the GBS group than in the control group (n = 477, MD = −1.98, 95% CI [−2.25, −1.71], P < 0.00001). The results showed significant heterogeneity in these studies (P = 0.05, I^2^ = 73%) (Figure 8A).

2. High-sensitivity CRP (hs-CRP)

Four studies reported hs-CRP. Random-effects models were used to analyze pooled data (n = 414, MD = −1.61, 95% CI [−2.66, −0.56], P = 0.003) with significant heterogeneity (P < 0.00001, I^2^ = 97%). Meta-analysis showed that the GBS preparations intervention group had a significantly greater reduction in hs-CRP than the control group (Figure 8B).

3. Tumor necrosis factor-α (TNF-α)

Two studies reported TNF-α, and no significant heterogeneity was observed among them (P = 0.80, I^2^ = 0%). A random-effects meta-analysis showed that TNF-α was significantly lower in the GBS intervention group. (n = 234, MD = −20.42, 95%CI [−32.58, −8.26], P = 0.0010) (Figure 8C).

##### 3.4.2.3 Efficacy on vascular function and endothelial regulation indicators

1. Matrix metalloproteinase-9 (MMP-9)

**FIGURE 9:**
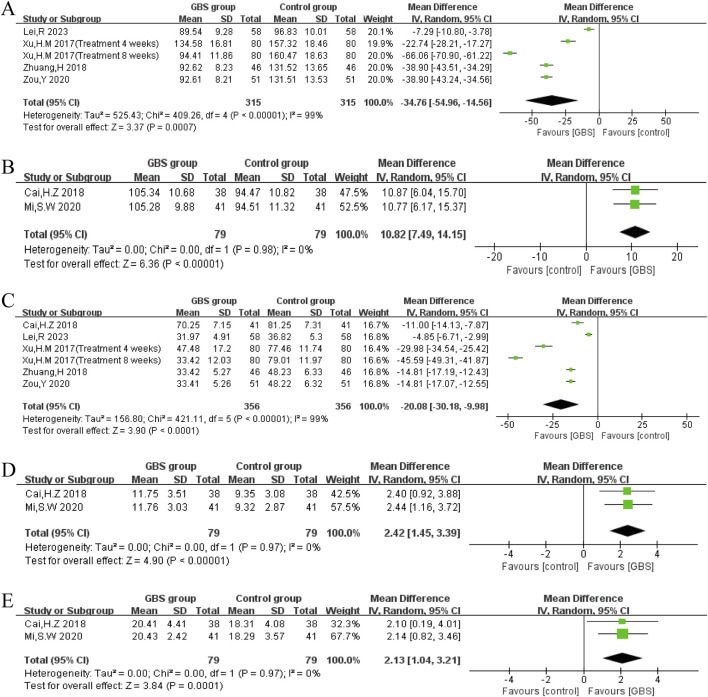
Effects of GBS on **(A)** MMP-9, **(B)** NO, **(C)** ET-1, **(D)** FMD, and **(E)** NMD.

MMP-9 was reported by four studies. It was significantly lower in the GBS preparations intervention group than in the control group (MD = −34.76, 95% CI [−54.96, −14.56], P = 0.0007). Meta-analysis under a random-effects model showed significant heterogeneity (P < 0.00001, I^2^ = 99%) (Figure 9A).

2. Nitric oxide (NO)

Two studies reported NO, and no significant heterogeneity was observed between them (P = 0.98, I^2^ = 0%). A random-effects model was used, and the meta-analysis showed that NO was higher in the GBS intervention group (n = 158, MD = 10.82, 95% CI [7.49, 14.15], P < 0.00001) (Figure 9B).

3. Endothelin-1 (ET-1)

ET-1 was reported in five studies. Meta-analysis showed that there was statistical difference between GBS intervention group in reducing ET-1 (MD = −20.08, 95% CI [−30.18, −9.98], P < 0.0001) for significant heterogeneity (P < 0.00001, I^2^ = 99%) (Figure 9C).

4. Flow-mediated dilation (FMD)

In two studies, FMD was reported. Random-effects models were used to analyze pooled data (n = 158, MD = 2.42, 95% CI [1.45, 3.39], P < 0.00001) with no significant heterogeneity (P = 0.97, I^2^ = 0%). Meta-analysis showed that the GBS group had a significantly greater increase in FMD than the control group (Figure 9D).

5. Nitroglycerin-mediated dilation (NMD)

NMD was reported in two studies, and no significant heterogeneity was observed among them (P = 0.97, I^2^ = 0%). A random-effects model was used, and the meta-analysis showed that NMD was higher in the GBS preparations intervention group (n = 158, MD = 2.13, 95% CI [1.04, 3.21], P = 0.0001) (Figure 9E).

##### 3.4.2.4 Efficacy on psychological state

1. Self-Rating Anxiety Scale (SAS)

**FIGURE 10:**
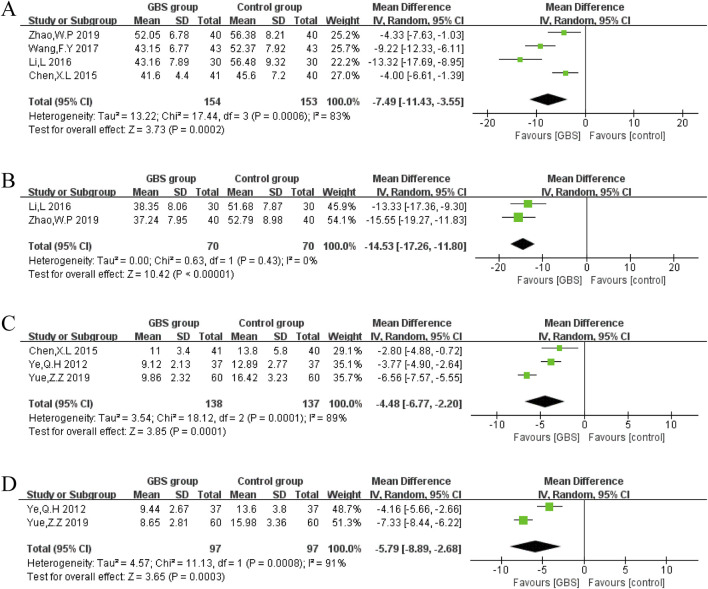
Forest plot of impacts of GBS on **(A)** SAS, **(B)** SDS, **(C)** HAMA, and **(D)** HAMD.

SAS was evaluated in four studies. A random-effects model was constructed, and the meta-analysis results showed that SAS was lower in the GBS intervention group than in the control group (n = 307, MD = −7.49, 95% CI [−11.43, −3.55], P = 0.0002). The results showed significant heterogeneity between these studies (P = 0.0006, I^2^ = 83%) (Figure 10A).

2. Self-Rating Depression Scale (SDS)

SDS was reported by two studies. It was significantly lower in the GBS group than in the control group (n = 140, MD = −14.53, 95% CI [−17.26, −11.80], P < 0.00001). Meta-analysis results showed no significant heterogeneity in two studies (P = 0.43, I^2^ = 0%) (Figure 10B).

3. Hamilton Anxiety Rating Scale (HAMA)

HAMA was reported in three studies. Random-effects models were constructed to analyze pooled data (n = 275, MD = −4.48, 95% CI [−6.77, −2.20], P = 0.0001) with significant heterogeneity (P = 0.0001, I2 = 89%) (Figure 10C). Compared with the control group, the meta-analysis showed that the GBS preparations intervention group had a significant decrease in HAMA.

4. Hamilton Depression Rating Scale (HAMD).

HAMD was evaluated in two trials. A random-effects model was used to analyze pooled data (n = 194, MD = −5.79, 95% CI [−8.89, −2.68], P = 0.0003) with a heterogeneous distribution (P = 0.0008, I2 = 91%) (Figure 10D). Based on the meta-analysis, GBS intervention was more beneficial in reducing HAMD scores in HF patients than in the control group.

### 3.5 Adverse events

Adverse events were documented in detail in 13 of the 32 included studies. Detailed information is provided in Table 1. The statistics of adverse events and their incidence were in both groups as follows: allergy (T: 3.33%; C: 6.67%), skin rash (T: 3.45%; C: 1.72%), drowsiness (T: 3.45%; C: 1.72%), fatigue (T: 3.6%; C: 14.4%), dry mouth (T: 24.32%; C: 29.73%), insomnia (T: 5.41%; C: 29.73%), dizziness and/or headache (T: 7.51%; C: 15.05%), blurred vision (T: 8.11%; C: 13.51%), nausea and/or vomiting (T: 5.85%; C: 8.9%), stomach upset (T: 6.35%; C: 7.94%), abdominal distension (T: 2.96%; C: 12.18%), abdominal pain and diarrhea (T: 3.45%; C: 3.45%), constipation (T: 13.51%; C: 16.22%), palpitations and chest tightness (T: 10.45%; C: 26.87%), arrhythmia (T: 2.25%; C: 15.73%), slowing of the heart rate or bradycardia (T: 1.74%; C: 0.87%), hepatic abnormalities or elevated transaminases (T: 2.24%; C: 9.77%), hypotension (T: 1.92%; C: 3.85%), hypertension (T: 5.41%; C: 10.81%), and electrolyte disorders (T: 0%; C: 1.92%). Among these, the incidence of adverse events was generally similar or lower in the GBS add-on therapy group than in the conventional therapy alone group, but a causal relationship remains unclear. Moreover, the fact that 19 trials did not provide detailed safety reports further limits our ability to draw definitive safety conclusions.

### 3.6 Sensitivity analysis

Sensitivity analyses were performed using the leave-one-out method. Significant heterogeneity was identified in the meta-analyses of LVEF, LVEDD, LVESD, BNP, NT-proBNP, 6MWTD, LVPWT, CRP, hs-CRP, CI, CO, MMP-9, ET-1, HAMA, and HAMD. In contrast, heterogeneity was non-significant for TNF-α, FS, SV, NO, FMD, NMD, SAS, and SDS outcomes. The leave-one-out analysis revealed that: (1) Removal of one study (He, 2015) in the CI analysis reduced heterogeneity from 59% to 0%. This may be attributable to the substantially greater MD in this study compared to others. (2) Exclusion of one study (Yue et al., 2019) in the HAMA analysis decreased heterogeneity from 89% to 0%, potentially due to this trial’s larger effect size.

### 3.7 Publication bias

**FIGURE 11:**
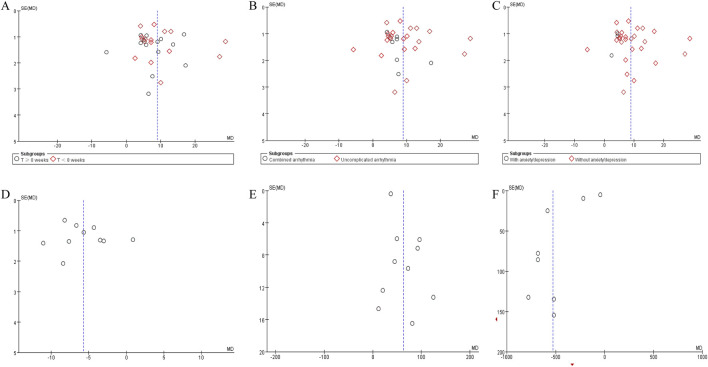
Funnel plot of **(A)** LVEF subgroups by treatment duration, **(B)** LVEF subgroups by with or without arrhythmia, **(C)** LVEF subgroups by with or without anxiety/depression, **(D)** LVEDD, **(E)** 6MWTD, and **(F)** NT-proBNP.

Funnel plots were generated to assess potential publication bias for the following outcomes: LVEF stratified by subgroup analysis, LVEDD, 6MWTD, and NT-proBNP (Figure 11). Egger’s linear regression test was performed to quantitatively evaluate funnel plot asymmetry. The results indicated no significant publication bias for LVEF (intercept = −5.52, 95%CI [-12.64, 1.60], P = 0.124) and LVEDD (intercept = −2.04, 95%CI [-9.53, 5.45], P = 0.553). In contrast, significant asymmetry was detected for NT-proBNP (intercept = 17.14, 95%CI [2.80, 31.48], P = 0.024) or 6MWTD (intercept = −13.18, 95%CI [-25.52, −0.83], P = 0.039). Given the asymmetric funnel plots and significant Egger test results for NT-proBNP and 6MWTD, these findings should be interpreted with caution, as publication bias may have inflated the effect estimates.

### 3.8 GRADE rating

We assessed the certainty of evidence using the GRADE framework for 23 outcomes. Due to severe methodological limitations, potential publication bias, and substantial heterogeneity among included trials, the overall evidence quality was low. The certainty of TNF-α, FS, and SV was moderate, while the certainty of LVEF, LVEDD, LVESD, BNP, NT-proBNP, 6MWTD, CRP, hs-CRP, CI, CO, MMP-9, ET-1, HAMA, and SAS was low. Additionally, the certainty of evidence was very low for LVPWT, NO, FMD, NMD, SDS, and HAMD (Table 2).

**TABLE 2:** Quality of evidence.

Quality assessment							No of patients		Effect		Quality	Importance
No of studies	Design	Risk of bias	Inconsistency	Indirectness	Imprecision	Other considerations	GBS	Control	Relative (95% CI)	Absolute
LVEF (better indicated by higher values)												
29	Randomised trials	Serious	Serious	No serious indirectness	No serious imprecision	None	1,678	1,670	-	MD 8.91 higher (6.78–11.04 higher)	⊕⊕ΟΟ LOW	CRITICAL
LVEDD (better indicated by lower values)												
10	Randomised trials	Serious	Serious	No serious indirectness	No serious imprecision	None	619	614	-	MD 5.71 lower (7.59–3.82 lower)	⊕⊕ΟΟ LOW	CRITICAL
LVESD (better indicated by lower values)												
6	Randomised trials	Serious	Serious	No serious indirectness	No serious imprecision	None	401	396	-	MD 6.30 lower (10–2.59 lower)	⊕⊕ΟΟ LOW	CRITICAL
LVPWT (better indicated by lower values)												
2	Randomised trials	Serious	Serious	No serious indirectness	Serious	None	116	116	-	MD 1.50 lower (3.09 lower to 0.09 higher)	⊕ΟΟΟ VERY LOW	IMPORTANT
BNP (better indicated by lower values)												
7	Randomised trials	Serious	Serious	No serious indirectness	No serious imprecision	None	571	566	-	MD 159.86 lower (199.17–120.56 lower)	⊕⊕ΟΟ LOW	CRITICAL
NT-proBNP (better indicated by lower values)												
10	Randomised trials	Serious	Serious	No serious indirectness	No serious imprecision	None	499	497	-	MD 529.13 lower (673.92–384.33 lower)	⊕⊕ΟΟ LOW	CRITICAL
6MWTD (better indicated by higher values)												
9	Randomised trials	Serious	Serious	No serious indirectness	No serious imprecision	None	683	678	-	MD 63.11 higher (43.27–82.95 higher)	⊕⊕ΟΟ LOW	CRITICAL
CRP (better indicated by lower values)												
2	Randomised trials	Serious	Serious	No serious indirectness	No serious imprecision	None	239	238	-	MD 1.98 lower (2.25–1.71 lower)	⊕⊕ΟΟ LOW	IMPORTANT
Hs-CRP (better indicated by lower values)												
4	Randomised trials	Serious	Serious	No serious indirectness	No serious imprecision	None	207	207	-	MD 1.61 lower (2.66–0.56 lower)	⊕⊕ΟΟ LOW	IMPORTANT
TNF-α (better indicated by lower values)												
2	Randomised trials	Serious	No serious inconsistency	No serious indirectness	No serious imprecision	None	117	117	-	MD 20.42 lower (32.58–8.56 lower)	⊕⊕⊕Ο MODERATE	IMPORTANT
FS (better indicated by higher values)												
4	Randomised trials	Serious	No serious inconsistency	No serious indirectness	No serious imprecision	None	194	194	-	MD 2.63 higher (2.04–3.22 higher)	⊕⊕⊕Ο MODERATE	IMPORTANT
SV (better indicated by higher values)												
4	Randomised trials	Serious	No serious inconsistency	No serious indirectness	No serious imprecision	None	231	230	-	MD 6.68 higher (5.56–7.8 higher)	⊕⊕⊕Ο MODERATE	IMPORTANT
CI (better indicated by higher values)												
3	Randomised trials	Serious	Serious	No serious indirectness	No serious imprecision	None	171	170	-	MD 0.51 higher (0.33–0.7 higher)	⊕⊕ΟΟ LOW	IMPORTANT
CO (better indicated by higher values)												
6	Randomised trials	Serious	Serious	No serious indirectness	No serious imprecision	None	317	315	-	MD 0.68 higher (0.38–0.99 higher)	⊕⊕ΟΟ LOW	IMPORTANT
MMP-9 (better indicated by lower values)												
4	Randomised trials	Serious	Serious	No serious indirectness	No serious imprecision	None	315	315	-	MD 34.76 lower (54.96–14.56 lower)	⊕⊕ΟΟ LOW	NOT IMPORTANT
NO (better indicated by higher values)												
2	Randomised trials	Serious	No serious inconsistency	No serious indirectness	Very serious	None	79	79	-	MD 10.82 higher (7.49–14.15 higher)	⊕ΟΟΟ VERY LOW	NOT IMPORTANT
ET-1 (better indicated by lower values)												
5	Randomised trials	Serious	Serious	No serious indirectness	No serious imprecision	None	356	356	-	MD 20.08 lower (30.18–9.98 lower)	⊕⊕ΟΟ LOW	NOT IMPORTANT
FMD (better indicated by higher values)												
2	Randomised trials	Serious	No serious inconsistency	No serious indirectness	Very serious	None	79	79	-	MD 2.42 higher (1.45–3.39 higher)	⊕ΟΟΟ VERY LOW	NOT IMPORTANT
NMD (better indicated by higher values)												
2	Randomised trials	Serious	No serious inconsistency	No serious indirectness	Very serious	None	79	79	-	MD 2.13 higher (1.04–3.21 higher)	⊕ΟΟΟ VERY LOW	NOT IMPORTANT
SAS (better indicated by lower values)												
4	Randomised trials	Serious	Serious	No serious indirectness	No serious imprecision	None	154	153	-	MD 7.49 lower (11.43–3.55 lower)	⊕⊕ΟΟ LOW	NOT IMPORTANT
SDS (better indicated by lower values)												
2	Randomised trials	Serious	No serious inconsistency	No serious indirectness	Very serious	None	70	70	-	MD 14.53 lower (17.26–11.8 lower)	⊕ΟΟΟ VERY LOW	NOT IMPORTANT
HAMA (better indicated by lower values)												
3	Randomised trials	Serious	Serious	No serious indirectness	No serious imprecision	None	138	137	-	MD 4.48 lower (6.77–2.2 lower)	⊕⊕ΟΟ LOW	NOT IMPORTANT
HAMD (better indicated by lower values)												
2	Randomised trials	Serious	Serious	No serious indirectness	Very serious	None	97	97	-	MD 5.79 lower (8.89–2.68 lower)	⊕ΟΟΟ VERY LOW	NOT IMPORTANT

## 4 Discussion

### 4.1 Summary of evidence

This systematic review synthesizes evidence from 32 RCTs (n = 3,476) evaluating the efficacy and safety of GBS for HF. The meta-analysis identified statistically significant associations between GBS supplementation and improvements in key surrogate cardiac function parameters, as well as in clinical symptoms and quality of life measures. However, these findings must be interpreted in the context of predominantly low or very low certainty evidence, the absence of data on hard clinical endpoints, and the substantial methodological limitations identified across the included trials.

Most notably, LVEF increased by an MD of 8.91% (95%CI [6.78, 11.04], P < 0.00001), accompanied by favorable reductions in ventricular remodeling markers (LVEDD, LVESD) and neurohormonal activation (BNP, NT-proBNP). Subgroup analyses stratified by treatment duration (T < 8 weeks, T ≥ 8 weeks) and presence of comorbid arrhythmias demonstrated persistent significant heterogeneity. In contrast, subgroup analysis based on comorbid anxiety and/or depression showed substantially reduced heterogeneity while maintaining significant therapeutic efficacy in the GBS intervention group. In addition, there was an improvement in cardiac function indices (FS, SV, CI, CO). Functional capacity, measured by 6MWTD, also showed clinically relevant enhancement. Mechanistically, these benefits align with GBS-mediated attenuation of inflammatory pathways, evidenced by significant reductions in CRP, hs-CRP, and TNF-α. Pooled data indicate meaningful improvements in psychological outcomes (SAS, SDS, HAMA, HAMD) and exercise capacity (6MWTD), suggesting a likely beneficial effect on health-related quality of life, although dedicated quality of life instruments were not used in these trials. Our analysis further indicates that GBS significantly modulates mediators of vascular homeostasis, demonstrating coherent effects across five endothelial regulation biomarkers (MMP-9, NO, ET-1, FMD, NMD). The marked reduction in MMP-9 aligns with GBS’s putative anti-fibrotic properties. Elevated MMP-9 drives extracellular matrix degradation in HF, accelerating ventricular remodeling (Deschamps and Spinale, 2006; Tsuruda et al., 2004). The observed 34.76-unit decrease in MMP-9 suggests a potential attenuation of myocardial fibrosis by GBS. However, extreme heterogeneity (P < 0.00001, I^2^ = 99%) necessitates cautious interpretation, as it likely reflects clinical variability in HF phenotypes (ischemic versus non-ischemic) or differential sampling timepoints relative to disease progression. NO elevation (MD = 10.82, P < 0.00001) and ET-1 reduction (MD = −20.08, P < 0.0001) indicate improved endothelial vasoregulatory function. FMD improvement confirms enhancement of endothelium-dependent vasodilation, directly linked to NO upregulation. And the NMD increase reflects smooth muscle responsiveness, suggesting GBS’s benefits extend beyond the endothelium to the vascular media. The findings of this study are consistent with our team’s previous research (Wang et al., 2025), confirming that adjunctive TCM therapy improves LVEF, mitigates cardiac remodeling, enhances cardiac function, and increases exercise tolerance in patients with HF, thereby significantly improving quality of life. Furthermore, this study extends these observations by demonstrating that GBS adjunctive therapy provides additional benefits, including improved psychological status, reduced inflammatory response, and protection of vascular endothelial function, which collectively contribute to a further enhancement in patient quality of life.

The safety profile of GBS remains uncertain. Although no major safety signals were directly attributed to GBS in the included studies, adverse event data were incompletely reported in 19 of 32 trials, and the lack of standardized safety monitoring limits any definitive conclusions. Therefore, while no immediate safety concerns were identified, the evidence is insufficient to establish a favorable safety profile.

### 4.2 Limitations

Our findings suggest that adjunctive GBS therapy may offer potential benefits in surrogate measures of cardiac function and patient-reported quality of life. Nevertheless, this systematic review is subject to several critical limitations that warrant cautious interpretation. Firstly, the exclusive inclusion of trials from China published in domestic journals introduces considerable geographical bias and potential publication selectivity, severely limiting the generalizability of our findings to other populations. Secondly, pervasive methodological weaknesses were identified across included studies. Although attrition was low for the reported outcomes (leading to a “low risk” rating in Domain 3 for all studies), some trials did not report certain pre-specified outcomes (e.g., long-term follow-up data), which limits the comprehensiveness of our analysis. The substantial heterogeneity observed may also stem from clinical differences in HF severity (encompassing NYHA classes II–IV), variations in concomitant background therapies, or the instability of estimates inherent to small sample sizes. Unfortunately, patient-level data were not available to conduct subgroup analyses based on etiology (e.g., ischemic/non-ischemic), HF phenotype (HFrEF, HFmrEF, HFpEF), age, or gender to further explore these potential effect modifiers. Thirdly, insufficient documentation of GBS-related adverse reactions precludes definitive safety conclusions, necessitating further safety evaluation. Fourth, publication bias assessment revealed significant asymmetry for NT-proBNP and 6MWTD, suggesting that smaller studies with positive results may be overrepresented in the literature. This potential publication bias may have led to overestimation of the true treatment effects. Additionally, since all intervention groups received GBS concomitantly with conventional medications, potential drug interactions confounding efficacy outcomes cannot be excluded. Most critically, the fundamental absence of data on hard clinical endpoints—specifically cardiovascular mortality, all-cause mortality, and heart failure rehospitalization rates—represents the most crucial limitation of our analysis. While improvements in surrogate markers like LVEF and 6MWTD are valuable for exploring mechanisms and short-term efficacy, they cannot be equated with a proven reduction in major adverse clinical events, which are paramount for clinical decision-making in heart failure. This omission precludes any definitive conclusions regarding the impact of GBS on disease progression and long-term patient prognosis. Collectively, these limitations resulted in low or very low GRADE certainty for 87% of outcomes, with funnel plot asymmetry further indicating potential publication bias.

### 4.3 Suggestions for future research

Although this systematic review and meta-analysis indicate potential therapeutic benefits of GBS for HF, substantial heterogeneity in key outcomes and methodological limitations inherent in the included trials necessitate cautious interpretation of these findings. To provide reliable evidence for clinical decision making, future trials should implement several key design improvements. Additionally, to mitigate geographical bias and enhance the generalizability of future findings, we strongly recommend that subsequent trials be international and multicenter in scope; such trials should also be high-quality, featuring large sample sizes, double-blinding, and placebo controls to rigorously validate the efficacy and safety of GBS in HF management. At the same time, to address the limitations identified in the GRADE assessment and to enhance the quality of evidence from future research, we propose that upcoming clinical studies prioritize stringent methodological designs. Employing rigorous study designs with adequate blinding, allocation concealment, placebo controls, and pre-registration of trial protocols is paramount. It is crucial that all trials, including those with neutral or negative findings, be prospectively registered and that their results be made publicly available to mitigate publication bias. Concurrent pharmacodynamic evaluations should assess interactions between GBS and conventional HF medications, establishing causal attribution for adverse events through stratified treatment arms. Most critically, trials should prioritize time-to-event analyses of cardiovascular mortality and HF hospitalizations, supplemented by validated quality of life metrics such as the Kansas City Cardiomyopathy Questionnaire (KCCQ) (Green et al., 2000; Spertus et al., 2020). Furthermore, Mechanistic substudies exploring GBS’s effects on myocardial energetics and autonomic regulation would elucidate its cardioprotective pathways. Only through such methodologically robust investigations can we determine whether the observed physiological improvements translate into clinically meaningful benefits and definitively establish safety profiles. A prospective, multicenter, randomized controlled study evaluating the clinical efficacy of GBS (Zhenyuan Capsule) in the treatment of HF after myocardial infarction is currently available on the International Clinical Trials Registry Platform (https://trialsearch.who.int/Trial2.aspx?TrialID=ChiCTR2200057496). We will continue to monitor this study.

## 5 Conclusion

This systematic review provides preliminary, hypothesis-generating evidence suggesting that adjunctive GBS therapy may be associated with improvements in surrogate measures of cardiac function and patient-reported outcomes in HF. However, these findings are derived predominantly from low- and very low-certainty evidence, with no available data on hard clinical endpoints such as cardiovascular mortality or HF hospitalization. The significant methodological limitations, geographical bias, and substantial heterogeneity across included trials preclude any definitive conclusions regarding the efficacy or safety of GBS. Consequently, the current evidence base is insufficient to support the routine clinical use of GBS in HF management. Individualized application, if considered, should be undertaken with full acknowledgment of the underlying evidence uncertainty and in the context of shared decision-making. These findings underscore the urgent need for rigorous, multicenter, long-term randomized controlled trials designed to evaluate patient-important outcomes and establish whether GBS confers clinically meaningful benefits in well-defined HF populations.

## Data Availability

The original contributions presented in the study are included in the article/Supplementary Material, further inquiries can be directed to the corresponding authors.

## References

[B1] Attele A. S. Zhou Y. P. Xie J. T. Wu J. A. Zhang L. Dey L. (2002). Antidiabetic effects of Panax ginseng berry extract and the identification of an effective component. Diabetes 51 (6), 1851–1858. 10.2337/diabetes.51.6.1851 12031973

[B2] Bai Y. Cha L. Wang Y. Song Y. Y. Sun T. Du Y. Z. (2025). Progress of ginseng food deep-processing research and current status of industrialization. 37 (03), 81–87.

[B3] Balshem H. Helfand M. Schünemann H. J. Oxman A. D. Kunz R. Brozek J. (2011). GRADE guidelines: 3. Rating the quality of evidence. J. Clinical Epidemiology 64 (4), 401–406. 10.1016/j.jclinepi.2010.07.015 21208779

[B4] Bozkurt B. Coats A. J. S. Tsutsui H. Abdelhamid C. M. Adamopoulos S. Albert N. (2021). Universal definition and classification of heart failure: a report of the heart failure society of America, heart failure association of the european society of cardiology, Japanese heart failure society and writing committee of the universal definition of heart failure: endorsed by the Canadian heart failure society, heart failure association of India, cardiac society of Australia and New Zealand, and Chinese heart failure association. Eur. Journal Heart Failure 23 (3), 352–380. 10.1002/ejhf.2115 33605000

[B5] Cai H. Z. Xu Z. L. Liao L. Y. Tian M. Y. Chen S. H. (2018). The effects of total saponins from Panax ginseng on endothelial cell function in patients with chronic heart failure and ventricular arrhythmia. Chin. J. Clin. Healthc. 21 (6), 731–734. 10.3969/J.issn.1672-6790.2018.06.003

[B6] Cao F. Huang J. W. Liu P. (2017). Clinical study of zhenyuan capsules combined with adenosine monophosphate in treatment of chronic heart failure of coronary heart disease drugs & clinic 32 (11), 2120–2123. 28285900

[B7] Chandra A. Vaduganathan M. Lewis E. F. Claggett B. L. Rizkala A. R. Wang W. (2019). Health-related quality of life in heart failure with preserved ejection fraction: the PARAGON-HF trial. JACC. Heart Failure 7 (10), 862–874. 10.1016/j.jchf.2019.05.015 31302043

[B8] Chen C. G. (2014). Clinical observation on 45 cases of chronic heart failure treated with zhenyuan capsule. Guid. J. Traditional Chin. Med. Pharmacol. 20 (6), 106–107.

[B9] Chen X. L. Luo Z. Wei L. H. (2015). Clinical efficiency of zhenyuan capsule combined with small-dose lorazepam in treatment of elderly chronic heart failure patients with anxiety symptoms. Chin. J. Multiple Organ Dis. Elder. 14 (8), 602–606.

[B10] Chen X. Li Q. S. Zhang A. L. Zhong Q. F. (2021). Zhenyuan capsule combined with trimetazidine in the treatment of coronary heart disease combined with chronic heart failure effectiveness in patients with chronic heart failure. Med. J. Chin. People's Health 33 (13), 70–72.

[B11] Choi H. S. Kim S. Kim M. J. Kim M. S. Kim J. Park C. W. (2018). Efficacy and safety of Panax ginseng berry extract on glycemic control: a 12-wk randomized, double-blind, and placebo-controlled clinical trial. J. Ginseng Research 42 (1), 90–97. 10.1016/j.jgr.2017.01.003 29348727 PMC5766700

[B12] Conrad N. Judge A. Tran J. Mohseni H. Hedgecott D. Crespillo A. P. (2018). Temporal trends and patterns in heart failure incidence: a population-based study of 4 million individuals. Lancet London, Engl. 391 (10120), 572–580. 10.1016/S0140-6736(17)32520-5 29174292 PMC5814791

[B13] Dai C. J. (2013). Clinical observation of zhengyuan capsule on patients both with coronary heart disease heart failure and impaired glucose regulation. CHINA Pract. Med. 8 (24), 22–23.

[B14] Deschamps A. M. Spinale F. G. (2006). Pathways of matrix metalloproteinase induction in heart failure: bioactive molecules and transcriptional regulation. Cardiovasc. Research 69 (3), 666–676. 10.1016/j.cardiores.2005.10.004 16426590

[B15] Diseases S. (2022). Guidelines for the clinical use of proprietary Chinese medicines in the treatment of heart failure (2021). Chin. J. Integr. Traditional West. Med. 42 (03), 261–275.

[B16] Duan X. Z. Wang X. Y. Xu J. Wang W. Liu W. W. (2014). Zhenyuan capsule treatment of coronary heart disease chronic heart failure 42 cases efficacy observation. Chin. J. Traditional Med. Sci. Technol. 21 (2), 192–193.

[B17] F Z. H. Mi H. Qu X. Y. Pang W. Guo Y. T. (2013). Research on HPLC fingerprint in fruit of *Panax ginseng* . Special Wild Econ. Animal Plant Res. 35 (04), 30–33.

[B18] Fan H. J. Li Z. Y. Kang K. N. Ren W. Y. An W. Q. (2023). Clinical study on zhenyuan capsules combined with sacubitril valsartan in treatment of chronic heart failure. Drugs & Clin. 38 (5), 1132–1136.

[B19] Gao J. Shi J. Ma X. Lu F. Fu C. Chen Z. (2024). Effects of ginseng berry saponins from panax ginseng on glucose metabolism of patients with prediabetes: a randomized, double-blinded, placebo-controlled, crossover trial. Phytomedicine International Journal Phytotherapy Phytopharmacology 132, 155842. 10.1016/j.phymed.2024.155842 39004031

[B20] Green C. P. Porter C. B. Bresnahan D. R. Spertus J. A. (2000). Development and evaluation of the Kansas City cardiomyopathy questionnaire: a new health status measure for heart failure. J. Am. Coll. Cardiol. 35 (5), 1245–1255. 10.1016/s0735-1097(00)00531-3 10758967

[B21] Greene S. J. Butler J. Albert N. M. DeVore A. D. Sharma P. P. Duffy C. I. (2018). Medical therapy for heart failure with reduced ejection fraction: the CHAMP-HF registry. J. Am. Coll. Cardiol. 72 (4), 351–366. 10.1016/j.jacc.2018.04.070 30025570

[B22] Guyatt G. H. Oxman A. D. Kunz R. Atkins D. Brozek J. Vist G. (2011). GRADE guidelines: 2. Framing the question and deciding on important outcomes. J. Clinical Epidemiology 64 (4), 395–400. 10.1016/j.jclinepi.2010.09.012 21194891

[B23] He P. H. (2015). Effects of zhenyuan capsule on cardiac function and hs-CRP, NT-proBNP in patients with chronic heart failure. Laboratory Med. Clin. z2, 129–132. 10.3969/j.issn.1672-9455.2015.26.056

[B24] Heidenreich P. A. Albert N. M. Allen L. A. Bluemke D. A. Butler J. Fonarow G. C. (2013). Forecasting the impact of heart failure in the United States: a policy statement from the American heart association. Circ. Heart Failure 6 (3), 606–619. 10.1161/HHF.0b013e318291329a 23616602 PMC3908895

[B25] Heidenreich P. A. Bozkurt B. Aguilar D. Allen L. A. Byun J. J. Colvin M. M. (2022). 2022 AHA/ACC/HFSA guideline for the management of heart failure: a report of the American college of cardiology/american heart association joint committee on clinical practice guidelines. J. Am. Coll. Cardiol. 79 (17), e263–e421. 10.1016/j.jacc.2021.12.012 35379503

[B26] Hou W. Wang Y. Zheng P. Cui R. (2020). Effects of ginseng on neurological disorders. Front. Cellular Neuroscience 14, 55. 10.3389/fncel.2020.00055 32265659 PMC7099600

[B27] Hu J. Z. Wang S. (2017). Clinical efficacy of meglumine cyclic adenylate combined with zhenyuan capsule in treatment of chronic heart failure. Med. J. Natl. Defending Forces Northwest China 38 (12), 812–815. 10.16021/j.cnki.1007-8622.2017.12.012

[B28] Hu J. R. Chun Y. S. Kim J. K. Cho I. J. Ku S. K. (2019). Ginseng berry aqueous extract prevents scopolamine-induced memory impairment in mice. Exp. Therapeutic Medicine 18 (6), 4388–4396. 10.3892/etm.2019.8090 31772634 PMC6862129

[B29] Huang H. X. Li F. X. (2019). Clinical observation on zhenyuan capsule for the treatment of chronic heart failure in rheumatic heart disease. World Latest Medicne Inf. 19 (02), 114–115.

[B30] Lei R. Yin S. Diao Z. J. (2023). Effect of calcium dibutyryladenosine cyclophosphate combined with ginseng fruit saponins zhenyuan Capsule in the treatment of chronic heart failure complicated with arrhythmia. Chin. J. Drug Abuse Prev. Treat. 29 (5), 889–893.

[B31] Li D. F. (2009). Observation on the therapeutic effect of Zhenyuan capsule in treating chronic congestive heart failure with qi deficiency and blood stasis evidence. Hebei J. Of Traditional Chin. Med. 31 (11), 1703–1704.

[B32] Li K. Qi L. L. Li Y. H. (2014). Effect of zhenyuan capsule supplemented with comfort care on the treatment of chronic heart failure. China J. Pharm. Econ. 9 (6), 205–206.

[B33] Li L. Du H. Y. Li L. Z. Dai Y. Sun Y. (2016). Zhenyuan capsule treatment of coronary heart disease chronic heart failure with clinical efficacy analysis of patients with anxiety and depression state. Chin. J. Integr. Med. Cardio 14 (12), 1319–1321.

[B34] Li Z. Kim H. J. Park M. S. Ji G. E. (2018). Effects of fermented ginseng root and ginseng berry on obesity and lipid metabolism in mice fed a high-fat diet. J. Ginseng Research 42 (3), 312–319. 10.1016/j.jgr.2017.04.001 29983612 PMC6026359

[B35] Li W. Wang Y. Liu W. (2021). Research progress on development and utilization of non-medicinal parts of Panax ginseng and Panax quinquefolium. J. Jilin Agric. Univ. 43 (04), 383–392.

[B36] Liao Y. X. (2009). Clinical observation of effect of zhenyuan capsule on chronic heart failure. Chin. Tradit. Pat. Med. 31 (2), 179–181.

[B37] Lin Y. Fu S. Yao Y. Li Y. Zhao Y. Luo L. (2021). Heart failure with preserved ejection fraction based on aging and comorbidities. J. Transl. Med. 19 (1), 291. 10.1186/s12967-021-02935-x 34229717 PMC8259336

[B38] Liu Z. G. Wang X. M. Zhang J. L. (2011). Intervention study of zhenyuan capsule to 52 patients with asymptomatic heart failure. China Pract. Med. 6 (12), 36–38.

[B39] Liu M. Y. Ren Y. P. Zhang L. J. Ding J. Y. (2016). Pretreatment with ginseng fruit saponins affects serotonin expression in an experimental comorbidity model of myocardial infarction and depression. Aging Disease 7 (6), 680–686. 10.14336/AD.2016.0729 28053817 PMC5198858

[B40] Liu M. Liu J. Zhang L. Geng Q. Ge Y. (2019). Antidepressant-like effects of ginseng fruit saponin in myocardial infarction mice. Biomed. & Pharmacotherapy 115, 108900. 10.1016/j.biopha.2019.108900 31054510

[B41] Ma S. Hu J. Q. (2015). Observations on 36 cases of chronic heart failure treated with combination of Chinese and Western medicine. J. Pract. Traditional Chin. Med. 31 (10), 936–937.

[B42] Ma Z. N. Li Y. Z. Li W. Yan X. T. Yang G. Zhang J. (2017). Nephroprotective effects of saponins from leaves of Panax quinquefolius against cisplatin-induced acute kidney injury. Int. J. Mol. Sci. 18 (7). 10.3390/ijms18071407 28703736 PMC5535899

[B43] McDonagh T. A. Metra M. Adamo M. Gardner R. S. Baumbach A. Böhm M. (2021). 2021 ESC guidelines for the diagnosis and treatment of acute and chronic heart failure. Eur. Heart Journal 42 (36), 3599–3726. 10.1093/eurheartj/ehab368 34447992

[B44] McMurray J. J. Packer M. Desai A. S. Gong J. Lefkowitz M. P. Rizkala A. R. (2014). Angiotensin-neprilysin inhibition versus enalapril in heart failure. N Engl J Med. 371 (11), 993–1004. 10.1056/NEJMoa1409077 25176015

[B45] Mi S. W. (2020). Evaluation of the efficacy of chronic heart failure with ventricular arrhythmias treated with ginseng berry saponin. Chin. J. Conval. Med. 29 (6), 659–661.

[B46] Miao L. (2019). Efficacy of combined Chinese and Western medicine in the treatment of coronary heart failure and the effect on cardiac function. Shenzhen J. Integr. Traditional Chin. West. Med. 29 (18), 20–22.

[B47] Page M. J. McKenzie J. E. Bossuyt P. M. Boutron I. Hoffmann T. C. Mulrow C. D. (2021). The PRISMA 2020 statement: an updated guideline for reporting systematic reviews. BMJ Clin. Research ed. 372, n71. 10.1136/bmj.n71 33782057 PMC8005924

[B48] Pan S. Wang X. H. Ding H. W. (2019). Research and thoughts on ginseng industry. Ginseng Res. 31 (02), 55–58. 10.19403/j.cnki.1671-1521.2019.02.016

[B49] Rutledge T. Reis V. A. Linke S. E. Greenberg B. H. Mills P. J. (2006). Depression in heart failure a meta-analytic review of prevalence, intervention effects, and associations with clinical outcomes. J. Am. Coll. Cardiol. 48 (8), 1527–1537. 10.1016/j.jacc.2006.06.055 17045884

[B50] Savarese G. Becher P. M. Lund L. H. Seferovic P. Rosano G. M. C. Coats A. J. S. (2023). Global burden of heart failure: a comprehensive and updated review of epidemiology. Cardiovasc. Research 118 (17), 3272–3287. 10.1093/cvr/cvac013 35150240

[B51] Spertus J. A. Jones P. G. Sandhu A. T. Arnold S. V. (2020). Interpreting the Kansas City cardiomyopathy questionnaire in clinical trials and clinical care: JACC state-of-the-art review. J. Am. Coll. Cardiol. 76 (20), 2379–2390. 10.1016/j.jacc.2020.09.542 33183512

[B52] Tsuruda T. Costello-Boerrigter L. C. Burnett J. C. Jr. (2004). Matrix metalloproteinases: pathways of induction by bioactive molecules. Heart Failure Reviews 9 (1), 53–61. 10.1023/B:HREV.0000011394.34355.bb 14739768

[B53] Wang C. L. (2020). Zhenyuan capsules combined with trimetazidine on chronic heart failure with coronary heart disease. China Pharm. 29 (6), 116–118. 10.3969/j.issn.1004-2407.2018.06.033

[B54] Wang C. Z. Wu J. A. McEntee E. Yuan C. S. (2006). Saponins composition in American ginseng leaf and berry assayed by high-performance liquid chromatography. J. Agricultural Food Chemistry 54 (6), 2261–2266. 10.1021/jf052993w 16536605

[B55] Wang F. Y. Yuan Y. Ding S. Y. (2017). The efficacy of lorazepam combined with zhenyuan capsule in the treatment of chronic heart failure with anxiety symptoms. J. Clin. Res. 34 (12), 2410–2411.

[B56] Wang D. Lu Y. H. Zhai G. Q. Wang M. C. Yuan X. L. Jing H. Y. (2018). The effect of zhenyuan capsules combined with cyclic adenosine monophosphate on chronic heart failure and coronary heart disease. Northwest Pharm. J. 33 (6), 834–836. 10.3969/j.issn.1004-2407.2018.06.033

[B57] Wang J. Chang T. Liang Z. Cui Y. Wang X. Wang L. (2025). The efficacy and safety of Panax quinquefolius saponin for heart failure: a systematic review and meta-analysis. Front. Pharmacology 16, 1463609. 10.3389/fphar.2025.1463609 40093323 PMC11906658

[B58] Wen J. Wang C. M. Ding R. H. Liu Y. Ji C. Guo J. H. (2015). The efficacy of trimetazidine combined with zhenyuan capsule in the treatment of chronic heart failure. Hebei Med. J. 37 (9), 1367–1368.

[B59] Wu Q. Jia W. X. (2024). Effects of zhenyuan capsules combined with sacubitril valsartan sodium tablets on cardiac function and quality of life in patients with chronic heart failure. Clin. Res. Pract. 9 (27), 30–33. 10.19347/j.cnki.2096-1413.202427008

[B60] Xing J. J. Hou J. G. Liu Y. Zhang R. B. Jiang S. Ren S. (2019). Supplementation of saponins from leaves of Panax quinquefolius mitigates cisplatin-evoked cardiotoxicity via inhibiting oxidative stress-associated inflammation and apoptosis in mice. Antioxidants Basel, Switz. 8 (9). 10.3390/antiox8090347 31480577 PMC6769973

[B61] Xu H. M. Zhang J. Z. Zhang L. Guo G. L. Chang H. Gong L. C. (2017). Efficacy of ginseng berry saponin combined with calcium dibutyryl cyclophosphoryl adenosine in the treatment of chronic heart failure combined with slow-type arrhythmia. Chin. J. Gerontology 37 (6), 1384–1386.

[B62] Ye Q. H. Chen Z. B. Tang K. Pan H. B. Liu S. J. (2012). The combined treatment of zhenyuan capsule with paroxitine for anxiety and depression symptoms of chronic cardiac failure patients. Guangdong Med. J. 33 (12), 1820–1823.

[B63] Yue Z. Z. He S. N. Jiao Y. M. (2019). Effect of zhenyuan capsule combined with psychological intervention on efficacy safety and negative emotions of chronic heart failure. Shaanxi J. Traditional Chin. Med. 40 (3), 300–303.

[B64] Zhang R. S. Zhang Y. Zhou S. G. (2018). The effect of xinyuan capsule combined with zhenyuan capsule on chronic congestive heart failure cardiac function and exercise tolerance in patients with chronic congestive heart failure. China Health Care & Nutr. 28 (32), 86. 10.3969/j.issn.1004-7484.2018.32.126

[B65] Zhao W. P. Zhang X. Q. Zhang W. (2019). Analysis of the clinical effect of zhenyuan capsule in the treatment of coronary heart disease chronic heart failure patients with anxiety and depression status. J. Front. Med. 9 (32), 212–213.

[B66] Zhuang H. Jiang W. Yao F. Zou Y. Liu B. Huang L. (2018). Ginseng berry saponin combined with calcium dibutyryl cyclophosphate adenosine in the treatment of chronic heart failure combined with bradyarrhythmia efficacy observation. Mod. J. Integr. Traditional Chin. West. Med. 27 (32), 3612–3615.

[B67] Zou Y. Li L. (2020). Dibutyryl cyclophosphoryl adenosine calcium with ginseng fruit total saponin in the treatment of chronic heart failure combined with bradyarrhythmia. Chin. J. Integr. Med. Cardio 18 (14), 2293–2295.

