# Supplementary material for: The efficacy and safety of ginseng berry saponin for heart failure: a systematic review and meta-analysis
Source: Front Pharmacol. 2026 Apr 22;17:1712401. doi: 10.3389/fphar.2026.1712401 (PMC13144151; doi:10.3389/fphar.2026.1712401)
Supplement: Supplementary file 1 [file Supplementaryfile2.doc]

## Appendix B. Search Strategies for Chinese Databases

**CNKI 54**

(SU %= '心力衰竭' OR SU %= 'cardiac failure' OR SU %= 'Heart failure' OR SU %= '慢性心功能不全' OR SU %= '急性心功能不全' OR SU %= '急性心力衰竭' OR SU %= '慢性心力衰竭' OR SU %= '急性充血性心力衰竭' OR SU %= '慢性充血性心力衰竭' OR SU %= '慢性充血性心衰' OR SU %= '急性充血性心衰' OR SU %= '急性心衰' OR SU %= '慢性心衰' OR SU %= '充血性心衰' OR SU %= '充血性心力衰竭' OR SU %= '心功能不全' OR SU %= '心衰') AND ( SU %= '人参果总皂苷' OR SU %= '振源')

**VIP 54**

(U=心力衰竭 OR U=cardiac failure OR U=Heart failure OR U=慢性心功能不全 OR U=急性心功能不全 OR U=急性心力衰竭 OR U=慢性心力衰竭 OR U=急性充血性心力衰竭 OR U=慢性充血性心力衰竭 OR U=慢性充血性心衰 OR U=急性充血性心衰 OR U=急性心衰 OR U=慢性心衰 OR U=充血性心衰 OR U=充血性心力衰竭 OR U=心功能不全 OR U=心衰) AND (U=人参果总皂苷 OR U=振源)

**Wanfang Data 54**

(主题:(心力衰竭) or 主题:(cardiac failure) or 主题:(Heart failure) or 主题:(慢性心功能不全) or 主题:(急性心功能不全) or 主题:(急性心力衰竭) or 主题:(慢性心力衰竭) or 主题:(急性充血性心力衰竭) or 主题:(慢性充血性心力衰竭) or 主题:(慢性充血性心衰) or 主题:(急性充血性心衰) or 主题:(急性心衰) or 主题:(慢性心衰) or 主题:(充血性心衰) or 主题:(充血性心力衰竭) or 主题:(心功能不全) or 主题:(心衰)) and ( 主题:(人参果总皂苷) or 主题:("振源"))
